# Environmental stress reveals new insights regarding proteome rebalancing in *Arabidopsis thaliana* seeds

**DOI:** 10.1111/tpj.70881

**Published:** 2026-04-20

**Authors:** Huda Ansaf, Clement Bagaza, Abou Yobi, Thomas P. Mawhinney, Ruthie Angelovici

**Affiliations:** ^1^ Division of Biological Sciences, Interdisciplinary Plant Group, Christopher S. Bond Life Sciences Center University of Missouri Columbia Missouri 65211 USA; ^2^ Department of Biochemistry University of Missouri Columbia Missouri 65211 USA; ^3^ Department of Plant Biology Michigan State University East Lansing Michigan 48824 USA

**Keywords:** proteome rebalancing, seed storage proteins, amino acids, fatty acids, translation, reactive oxygen species, nitrogen, drought

## Abstract

Seeds are resilient to genetic and envirxonmental perturbation. For example, they can compensate for the loss of highly abundant seed storage proteins (SSPs) while maintaining amino acid levels and composition, a process known as proteome rebalancing. This buffering involves large‐scale adjustments in translational capacity and metabolic networks, yet it remains unclear whether such plasticity persists under environmental stress or eventually reaches a physiological limit. To address this, we examined Arabidopsis Col‐0 (wild type, WT) and *cruabc* (a triple SSP‐deficient mutant) dry seeds produced under no nitrogen supplementation, weekly nitrogen supplementation, or drought (water‐deficit) treatment imposed during the seed‐filling stage. Analyses of physiological traits, metabolic profiles, and proteomes revealed substantial treatment‐dependent changes; however, *cruabc* seeds consistently remained comparable to Col‐0 within each environment, indicating that rebalancing represents a robust, hard‐coded plasticity operating downstream of primary stress responses. Nevertheless, the molecular routes used to achieve metabolic and proteomic homeostasis differ across environments in rebalanced seeds. Under drought during seed filling, both genotypes upregulated photosynthetic and pentose phosphate pathway components to mitigate carbon limitation and energy stress, yet *cruabc* maintained a more oxidative redox state. Under low nitrogen, the *cruabc* dry seed proteome exhibited minimal reprogramming, whereas under high nitrogen, it underwent extensive remodeling, including enhanced translation‐related activity compared to WT. These findings suggest that proteome rebalancing represents a stable homeostatic endpoint that can be reset by environmental cues. However, the metabolic pathways and energetic costs required to achieve this state differ markedly between genotypes, revealing how SSP loss reshapes stress adaptation during seed maturation and desiccation. Collectively, our results refine the mechanistic framework underlying proteome rebalancing and establish a foundation for leveraging this process to enhance amino acid biofortification.

## Significance Statement

By demonstrating that genetic perturbations to seed storage proteins can be buffered across contrasting environmental conditions through coordinated adjustments in amino acid metabolism and the translational machinery, our work uncovers a key dimension governing proteome rebalancing. These findings address a long‐standing question in seed biology and have broad implications for improving seed nutritional quality and developing strategies for amino acid biofortification in crops under variable agricultural inputs.

## INTRODUCTION

Seed development is a highly coordinated process that progresses through two major phases, embryogenesis and maturation, with the maturation phase characterized by the accumulation of storage compounds and the acquisition of dormancy and desiccation tolerance (Baud et al., 2008). During embryogenesis, the embryo establishes polarity, basic tissue organization, and cell number (Goldberg et al., 1994). As development transitions into the reserve‐accumulation phase of maturation, metabolic activity intensifies to support biosynthesis of proteins, lipids, and carbohydrates (Baud et al., 2008). This seed‐filling stage was supported by elevated levels of translation, energy production, and plastid metabolism (Baud et al., 2008; Venglat et al., 2014; Verdier & Thompson, 2008). By desiccation, seeds reach a checkpoint of homeostasis where composition, storage reserves structure, and redox balance are optimized for long‐term viability (Nguyen et al., 2015; Ramtekey et al., 2022).

Seed maturation is defined by reserve accumulation, including the massive deposition of SSPs, which constitute 50%–70% of total seed protein in many cereals and oilseeds (Bagaza et al., 2024; Shewry & Halford, 2002). In dicots, 2S albumins and 12S globulins are the predominant SSPs (e.g., Arabidopsis, Brassica), whereas cereals are dominated by prolamins, including zeins in maize and kafirins in sorghum (Bagaza et al., 2025; Li et al., 2018; Shewry & Halford, 2002; Sjödahl et al., 1991). SSPs serve as major nitrogen and carbon sinks essential for early seedling growth and have been proposed to contribute to redox buffering and osmotic stability during late maturation (Bagaza et al., 2025; Nguyen et al., 2015; Verdier et al., 2013). SSPs are synthesized on the rough ER and, in the case of globulins such as Arabidopsis cruciferins, trafficked via the ER–Golgi– trans Golgi network (TGN) pathway to protein storage vacuoles, placing substantial demands on translational output, protein‐folding capacity, and endomembrane trafficking (Galili et al., 1993; Zheng et al., 2022). In contrast, prolamins aggregate within the ER lumen to form protein bodies and do not follow the canonical Golgi‐dependent route (Shimada et al., 2003; Zheng et al., 2022). Hormonal regulation tightly coordinates the onset of reserve deposition. During maturation, ABA levels rise sharply, activating a transcriptional program that promotes reserve accumulation, acquisition of desiccation tolerance, and dormancy (Ali et al., 2022; Rodríguez‐Gacio et al., 2009). ABA upregulates genes encoding SSPs and other maturation‐specific proteins such as late embryogenesis abundant (LEA) proteins. In contrast, GA antagonizes ABA, promotes growth, and reserves mobilization during germination (White & Rivin, 2000). The shift from a GA‐dominant to an ABA‐dominant hormonal environment terminates embryonic proliferation and triggers maturation (Yang et al., 2025).

Reserve accumulation in the seed requires a continuous supply of carbon and nitrogen from maternal tissues (Baud & Lepiniec, 2010; Weber et al., 2005). Carbon is transported largely in the form of sucrose, which is loaded into the phloem and unloaded into seed coat and endosperm tissues and cleaved into hexoses that fuel growth and reserve biosynthesis (Stein & Granot, 2019; Zhou et al., 2009). Early in seed development, high hexose levels correlate with cell division and expansion (Weber et al., 1995; Weber et al., 1997). During seed maturation, declining hexose and rising sucrose levels mark a shift from growth to storage and redirect metabolism toward reserve deposition, primarily oil in oilseeds and starch in cereals (Baud et al., 2008; Jenner et al., 1991). Nitrogen is delivered to the seed in the form of glutamine and asparagine and funneled into glutamate and downstream amino acids that support SSP synthesis (Masclaux‐Daubresse et al., 2010; Tegeder & Masclaux‐Daubresse, 2018). Because SSPs represent a major nitrogen sink in many seeds, nitrogen availability strongly influences reserve composition and content (Tabe et al., 2002).

Environmental stress significantly disrupts these carbon and nitrogen fluxes to the seeds. Drought (water deficit, WD) reduces stomatal conductance and photosynthetic capacity (Anjum et al., 2011), lowering sucrose export from source leaves, and constraining carbon delivery to seeds. This weakened source strength affects reproductive development, reducing grain set and seed filling through disrupted carbohydrate metabolism and hormonal imbalances. Drought increases non‐reducing sugars while suppressing starch accumulation, which can trigger ovary abortion in maize kernels (Andersen et al., 2002). Drought during seed filling greatly reduces yield by limiting carbon flux, impairing cell division, and reducing starch granule formation (Nicolas et al., 1985), which leads to yield reduction across legumes (Awasthi et al., 2014; Ghanbarı et al., 2013; Shrestha et al., 2006) and cereals (Araus et al., 2002). Reduction in glucose, fructose, and sucrose levels impairs sugar unloading from stems to seeds, leading to lower oil content and shifts in fatty acid composition (Bellaloui et al., 2013). For example, in maize, drought decreased oil content but increased linolenic and oleic acids (Ali et al., 2012), whereas in soybeans, it reduced oil content by up to 12.4% and decreased oleic acid levels (Dornbos Jr & Mullen, 1992). Several studies report increased relative seed protein under drought (Behboudian et al., 2001; Bouchereau et al., 1996; Gooding et al., 2003; Teixeira & Pereira, 2007), likely due to altered carbon partitioning and reduced seed size.

Nitrogen availability represents another major environmental driver in developing seeds. Nitrogen deficiency limits plant growth (Elser et al., 2007) as it is integral to proteins, nucleic acids, chlorophyll, and enzymes and key metabolic pathways (Robertson & Vitousek, 2009).

Under low N, plants show reduced leaf elongation (Lehmeier et al., 2013), decreased photosynthesis (Zhao et al., 2005), and smaller chloroplasts (Muller et al., 2009; Tang et al., 2019). Lower assimilate production consequently constrains carbon and nitrogen import into developing seeds. Low N limits the precursors required for SSP synthesis, while high N enhances N transport to seeds, increases SSP synthesis, and can reduce oil proportion due to the classic protein–oil trade‐off (Kambhampati et al., 2019).

Beyond environmental inputs, developing seeds also exhibit remarkable plasticity when their major nitrogen sinks, SSPs, are eliminated or strongly reduced. This phenomenon, known as proteome rebalancing, occurs when reduction or elimination of SSPs triggers extensive compensatory protein reprogramming, preserving total amino acid content and composition relative to wild type (Bagaza et al., 2024; Wu & Messing, 2014). Proteome rebalancing has been documented in maize (Morton et al., 2016), soybean (Schmidt et al., 2011), Arabidopsis thaliana (Bagaza et al., 2024), camelina (Schmidt & Pendarvis, 2017), and wheat (Altenbach et al., 2014), highlighting its strong conservation. However, because proteome rebalancing constrains changes in essential amino acid content and composition, it remains a central challenge for seed biofortification. Rebalancing is not a passive consequence of SSP loss but an actively regulated process. In Arabidopsis, elimination of major cruciferins alters the composition and abundance of ribosomal proteins and other translation‐related factors. This is consistent with the ribosomal heterogeneity model in which selective translation of alternative proteins compensates for the loss of highly abundant SSPs (Bagaza et al., 2024). SSP depletion in Arabidopsis also induced changes in redox and energy metabolism (Bagaza et al., 2024; Bagaza et al., 2025), reminiscent of canonical stress responses. For example, drought imposed during seed filling in Arabidopsis induces extensive proteome remodeling while preserving overall amino acid composition, suggesting that proteome rebalancing may integrate stress responses to maintain seed compositional homeostasis (Yobi et al., 2020).

In this study, we investigated how abiotic stress during seed filling shapes the final outcome of proteome rebalancing at the dry‐seed stage in *Arabidopsis thaliana* mutants lacking the three major 12S SSPs. We evaluated two contrasting stress conditions: (1) drought imposed during seed filling compared with well‐watered plants and (2) high versus low nitrogen availability imposed throughout seed filling. This experimental design allowed us to determine whether proteome rebalancing operates downstream of environmental stress responses.

Our findings demonstrate that proteome rebalancing operates downstream of environmental perturbations, resulting in a final seed composition that closely resembles that of stressed wild‐type plants. However, the SSP‐deficient mutant achieves this homeostasis through altered metabolic routes. Nitrogen availability strongly modulates how rebalancing is executed: under low nitrogen, the mutant proteome differs only modestly from the wild type, whereas under high nitrogen, it undergoes extensive proteomic remodeling. Together, these results indicate that proteome rebalancing is not a uniform process but is deployed in an environment‐dependent manner, with nitrogen availability exerting a particularly strong influence on compensatory mechanisms.

## RESULTS

### Seed composition is maintained following SSP reduction under variable water and nitrogen conditions

To determine whether eliminating SSPs alters seed traits under environmental stress, we compared seed yield, single seed weight, and seed composition between the *cruabc* triple mutant and Col‐0 across contrasting water and nitrogen conditions. Water‐stress treatments followed a previously published water stress during seed setting experiment (Yobi et al., 2020). Plants were grown under full‐watered (FW) and water‐deficit (WD) treatments, as well as under low‐ (LN) and high‐nitrogen (HN) supply, as described in the Materials and Methods. The *cruabc* mutant, which lacks the three major cruciferin SSPs (CRUA, CRUB, and CRUC), was generated by crossing crua‐Salk_00266, crub‐Salk_045987, and cruc‐GK_283D09, as originally described and validated by Withana‐Gamage et al. (2013) and previously characterized under well‐watered conditions (Bagaza et al., 2024).

#### Water treatment

**Figure 1:**
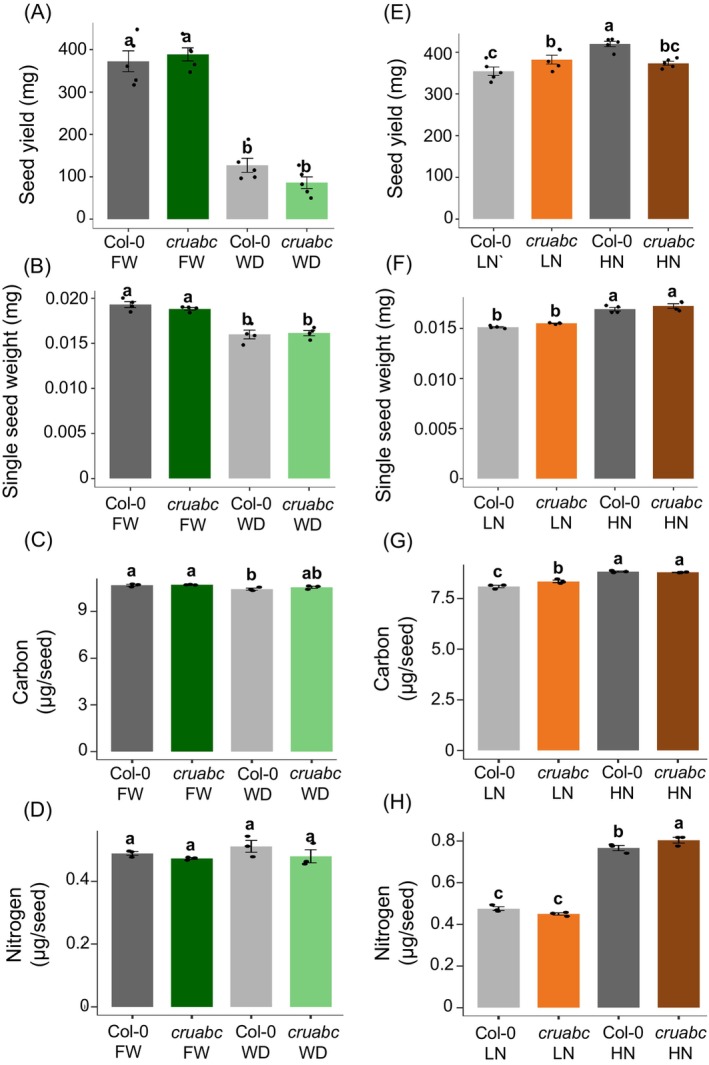
The effect of water deficit and contrasting nitrogen levels on seed yield, single seed weight, and carbon and nitrogen content in *Arabidopsis* cruciferin mutants (*cruabc*). (A–D) Water‐deficit treatment: The water deficit consists of withholding water from plants after initial bolting. (E–H) The nitrogen treatments consist of two concentrations: low nitrogen under which plants were watered once with the modified Bausenwein medium deprived of nitrogen for the rest of seed filling and maturation; high nitrogen under which plants were watered once with Bausenwein's medium, followed by watering 1 M ammonium nitrate once a week for the rest of seed filling and maturation. (A, E) Seed yield per plant. (B, F) Single seed weight. (C, G) Seed carbon content per seed. (D, H) Seed nitrogen content per seed. Error bars represent standard errors (*n* = 4 for seed weight, *n* = 5 for yield, *n* = 3 for elemental analysis). Different lowercase letters indicate statistically significant differences among genotypes according to Duncan's multiple range test (*P* < 0.05). Points represent individual biological replicates.

Under fully watered (FW), *cruabc*, and Col‐0 displayed comparable seed yield, seed weight, and carbon and nitrogen content per seed (Figure 1), consistent with proteome rebalancing. Under water deficit (WD), both genotypes exhibited a >3‐fold reduction in seed yield relative to FW, with no significant genotype differences (Figure 1A). Single seed weight also declined similarly in both lines (Figure 1B). Carbon and nitrogen content per seed remained indistinguishable between genotypes (Figure 1C,D).

#### Nitrogen treatment

Under low nitrogen (LN), *cruabc* showed slightly higher yield and higher carbon than Col‐0, while seed weight and nitrogen content were similar (Figure 1E–H). Under high nitrogen (HN), *cruabc* produced slightly lower yield and higher nitrogen relative to Col‐0, while seed weight and carbon were unchanged (Figure 1E–H). Interestingly, Col‐0 yield increased significantly from LN to HN, whereas *cruabc* yield did not (Figure 1E), indicating a genotype × nitrogen interaction in which SSP deficiency is advantageous under LN but not under HN.

Seed moisture content did not differ across treatments or genotypes (Table S1), confirming that observed weight differences reflect true dry mass variation rather than hydration differences. A representative SDS‐PAGE gel showing total seed protein profiles for all genotypes and treatments is presented in Figure S1.

### Protein‐bound amino acid composition is preserved across environmental conditions, while free amino acids are dynamically remodeled

To evaluate how water deficit or nitrogen availability affect protein‐bound (PBAA) and free amino acids (FAA) in dry seeds, we profiled PBAA and FAA pools in Col‐0 and *cruabc* grown under two water conditions (FW versus WD) and two nitrogen levels (LN versus HN), as described in Materials and Methods (Datas S1 and S2).

#### Water treatments

**Figure 2:**
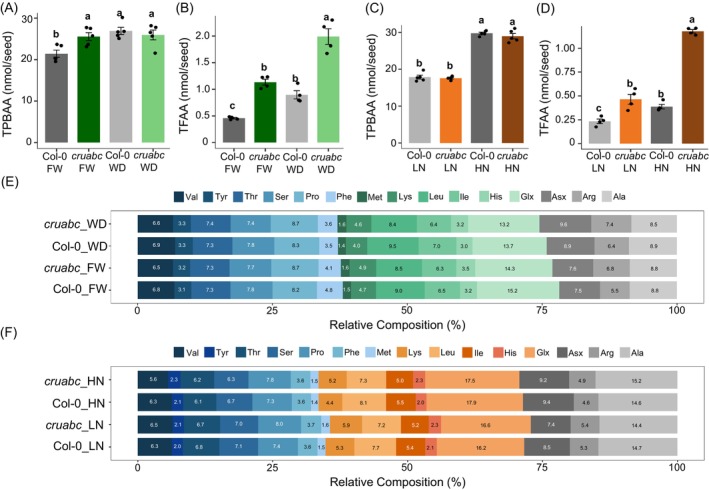
Protein‐bound amino acids (PBAAs) are rebalanced under both water deficit and nitrogen treatments in *Arabidopsis cruabc* mutant. (A) Total protein‐bound amino acids (TPBAAs) under full‐water (FW) and water‐deficit (WD) conditions for Col‐0 and *cruabc*. (B) Total free amino acids (TFAAs) under FW and WD conditions for Col‐0 and *cruabc*. (C) TPBAAs under low‐nitrogen (LN) and high‐nitrogen (HN) conditions for both genotypes. (D) TFAAs under LN and HN conditions for both genotypes. (E) 100% stacked barplots showing the relative composition of individual PBAAs under FW and WD for both genotypes. (F) 100% stacked barplots showing the relative composition of individual PBAAs under LN and HN for both genotypes. Error bars represent standard errors (*n* = 4 for FAA; *n* = 5 for PBAA). Different lowercase letters indicate statistically significant differences among genotypes according to Duncan's multiple range test (*P* < 0.05). Points represent individual biological replicates.

*Under FW*, total PBAAs (TPBAA) were slightly higher in *cruabc* than Col‐0 (Figure 2A; Tables S2A) despite equivalent seed nitrogen (Figure 1D), possibly reflecting shifts in unmeasured nitrogenous pools. Nevertheless, individual PBAA composition was largely preserved: 7 of 15 amino acids showed no difference under WD (Table S3A), and all shifts were <1.3% (Figure 2E). In contrast, total FAAs (TFAA) were ~2‐fold higher in *cruabc* (Figure 2B), with substantial compositional shifts (e.g., Asn increased by 18.28% and Glu decreased by 18.88%) (Table S3B).

*Under WD*, TPBAA increased similarly in both genotypes (Figure 2A; Tables S2A), likely due to smaller seeds and higher N per seed (Figure 1E–H). PBAA composition remained stable: 9 of 15 amino acids were unchanged (Table S3A), and all PBAA composition shifts < ~1.3% (Figure 2E; Table S3A). By contrast, TFAA increased substantially under WD in both genotypes, with an approximately twofold increase relative to their respective FW conditions (Figure 2B; Table S2B). Notably, due to its higher baseline, *cruabc* exhibited an overall ~4‐fold higher TFAA under WD compared to Col‐0 under FW. Under WD, Glu decreased the most (8.28%), while Lys (4.04%) and Thr (2.55%) increased the most (Table S2B). Differences in protein‐bound and free amino acids between *cruabc* and Col‐0 under FW and WD conditions are summarized in Tables S2 and S3.

#### Nitrogen treatments

*Under LN*, TPBAA in *cruabc* remained comparable to Col‐0 (Figure 2C; Table S4A). Only 5 of 15 individual PBAAs composition differed (Figure 2F; Table S5A). The composition of Asx, Ile, and Leu decreased at LN, while Lys and Met PBAA composition increased but no difference was greater than <1.2% (Table S5A). However, FAA composition had stronger shifts; Asn increased by 16.82% while Glu decreased by 8.19% (Table S5B).

*Under HN*, TPBAA remained similar between genotypes, confirming a highly buffered protein‐bound amino acid pool (Figure 2C). Five of 15 PBAAs shifted slightly (Figure 2F; Table S5A). At HN, His, Ile, Leu, and Ser all had lower PBAA composition while Lys increased. However, no PBAA composition differed by more than ~0.93% (Table S5A). FAA composition changed more dramatically: Arg and Asp increased by 12.79% and 12.27%, respectively, whereas Glu and Ser decreased by 8.65% and 4.57%, respectively (Table S5B), with Glu consistently reduced under both N conditions. Differences in protein‐bound and free amino acids between *cruabc* and Col‐0 under LN and HN conditions are summarized in Tables S4 and S5.

Overall, *cruabc's* PBAAs remain comparable to Col‐0 across environmental perturbations, whereas FAAs are strongly remodeled by SSP loss, drought, and nitrogen availability.

### Protein‐bound and free amino acid profiles show limited genotype × environment interactions

While total PBAA abundance and overall amino acid composition remain tightly buffered between genotypes across environments (Figure 2), we next asked whether subtle genotype × environment (G × E) effects could be detected for individual PBAAs and FAAs when examined at the level of absolute concentrations (nmol mg^−1^ dry seed).

#### Water treatments

**Figure 3:**
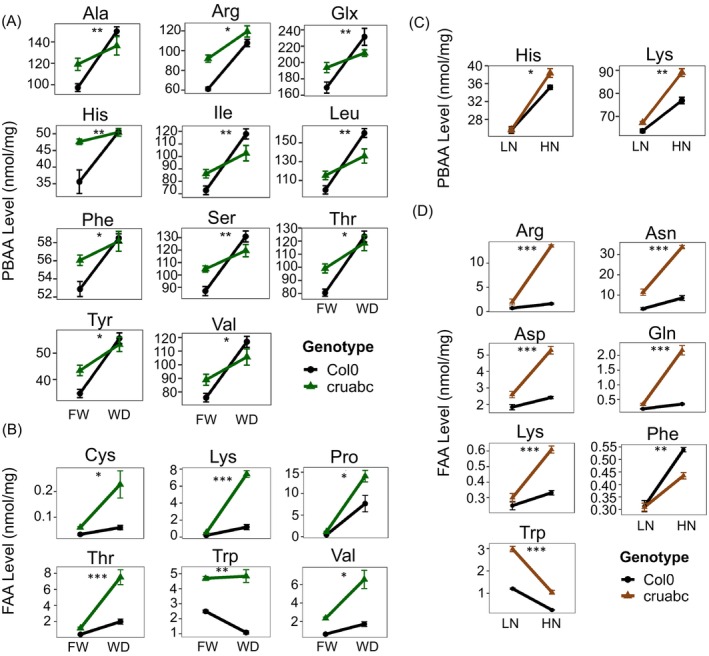
Significant genotype × environment interactions for free and protein‐bound amino acids (FAAs and PBAAs). (A, C) Protein‐bound amino acids (PBAAs), and (B, D) Free amino acids (FAAs) that exhibited significant genotype × treatment interaction effects (*P* < 0.05) from a two‐way ANOVA. Left panels (A, B) represent responses under water‐deficit treatment (FW versus WD), and right panels (C, D) represent responses under contrasting nitrogen treatments (LN versus HN). Each point represents the mean ± SE (*n* = 4–5) for each genotype. Asterisks denote the level of interaction significance: *P < 0.05, **P < 0.01, ***P < 0.001.

Under FW conditions, *cruabc* seeds displayed a slightly higher level of multiple PBAAs. However, these genotype differences diminished or in some cases reversed under WD, where PBAA levels in Col‐0 increased more than in *cruabc*. This convergence suggests that water‐deficit stress partially overrides genotype‐specific metabolic alterations in PBAA composition (Figure 3A). Importantly, these shifts are small in magnitude and do not alter the overall stability of the PBAA pool or its compositional balance between genotypes, consistent with robust buffering at the whole‐seed level. Because concentrations are expressed per mg dry seed, modest differences may also reflect treatment‐induced changes in seed size rather than true alterations in nitrogen allocation to protein. For FAAs, *cruabc* consistently accumulated higher levels than Col‐0 across water treatments (Table S2), although six FAAs showed significant G × E interactions under WD (Figure 3B). Under WD, both genotypes showed increased Pro and Val. Bound and free Thr and Val displayed significant genotype × treatment interactions, driven by stronger increases under WD. Non‐significant interactions for PBAAs and FAAs under water‐deficit conditions are shown in Figure S2.

#### Nitrogen treatments

PBAA concentrations generally increased under HN in both genotypes, consistent with enhanced protein synthesis per seed (Figure 3C); however, these shared increases occur within a globally buffered PBAA pool and do not alter overall compositional stability. Only two PBAAs—His and Lys—showed significant G × E interactions, with slightly higher concentrations in *cruabc* under HN. Thus, even under HN, PBAA responses are dominated by shared genotype‐independent increases, with only minor genotype‐specific deviations. In contrast, multiple FAAs exhibited strong G × E effects: *cruabc* showed pronounced HN‐induced increases in several nitrogen‐rich FAAs (e.g., Arg, Gln, Asn), whereas Col‐0 responded more moderately (Figure 3D). Non‐significant interactions for PBAAs and FAAs under nitrogen treatments can be found in Figure S3.

Together, these analyses indicate that genotype × environment interactions are far more prominent for FAAs than for PBAAs, reinforcing the conclusion that protein‐bound amino acids are globally buffered, whereas free amino acid pools provide the primary layer of metabolic flexibility.

### Total seed oil content is unchanged following SSP reduction, with minor shifts in fatty acid composition

**Figure 4:**
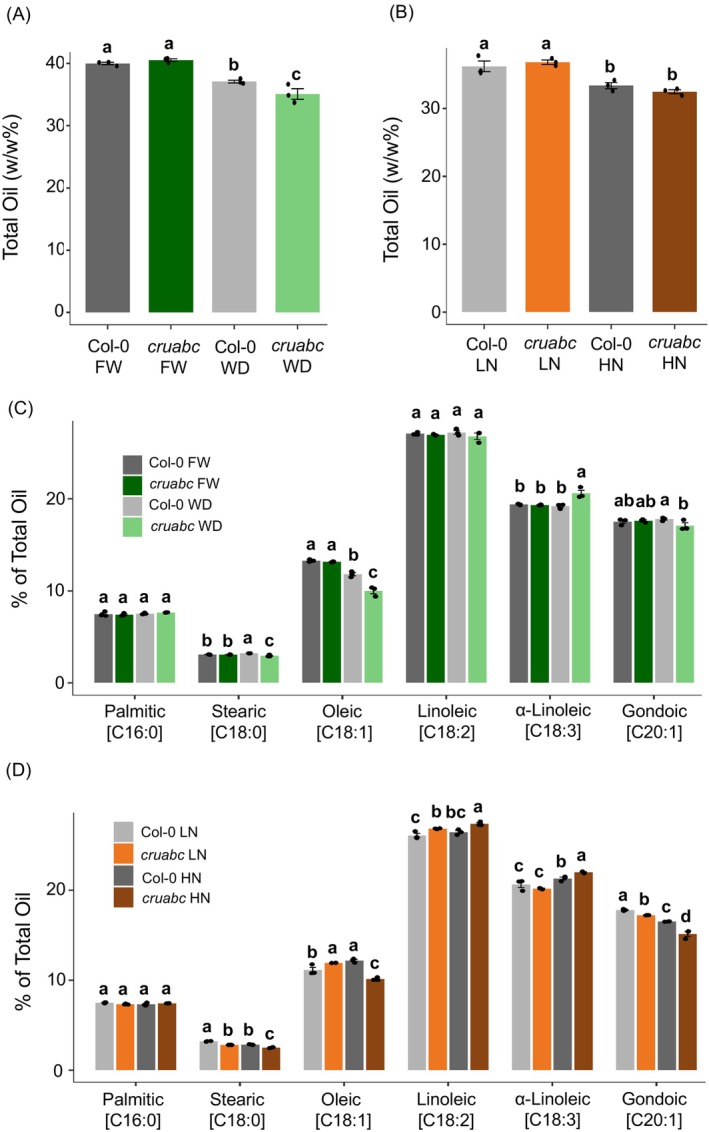
Total seed oil content and fatty acid composition in Col‐0 and *cruabc* under water and nitrogen treatments. (A) Total seed oil content (% dry weight) in Col‐0 and *cruabc* grown under full water (FW) and water deficit (WD). (B) Total seed oil content in Col‐0 and *cruabc* under low nitrogen (LN) and high nitrogen (HN). (C) Relative fatty acid composition (% of total oil) in Col‐0 and *cruabc* under water treatments (FW, WD). (D) Relative fatty acid composition under nitrogen treatments (LN, HN). Only the dominant fatty acids in Arabidopsis seeds are shown. Only the dominant fatty acids in Arabidopsis seeds are shown; all other detected fatty acids are provided in Data S3. Values are means ± SEM of biological replicates (*n* = 3). Points represent individual biological replicates.

Total seed oil (% dry weight per mg) and the relative abundance of the fatty acids (FAs) (% of total oil) were quantified in Col‐0 and *cruabc* under the various conditions. Comprehensive fatty acid profiles can be found in Data S3. Overall, SSP reduction primarily impacts fatty acid composition rather than total oil accumulation, with only modest genotype‐dependent effects on oil content across environments (Figure 4; Data S3).

#### Water treatments

*Under FW*, total oil content did not differ between genotypes (Figure 4A), and the major FA species (16:0, 18:0, 18:1, 18:2, 18:3, 20:1) were comparable (Figure 4C).

*Under WD*, total oil content decreased in both genotypes, with a stronger reduction in *cruabc* (Figure 4A). Several FAs also shifted specifically in *cruabc*: stearic (18:0), oleic (18:1), and gondoic/eicosenoic (20:1) decreased, whereas α‐linolenic (18:3) increased; other dominant FAs remained unchanged (Figure 4C). These changes suggest enhanced desaturation and reduced very long‐chain FA accumulation in *cruabc* under stress.

#### Nitrogen treatments

*At LN*, total oil content was similar between genotypes (Figure 4B). The *cruabc* mutant showed lower stearic acid (18:0) and gondoic/eicosenoic (20:1) but higher oleic acid (18:1) and linoleic acid (18:2) compared to Col‐0, along with modest increases in α‐linolenic (18:3). *At HN*, total oil content declined in both genotypes (Figure 4B). FA composition again diverged in a genotype‐dependent manner (Figure 4D): *cruabc* consistently exhibited lower 18:0, 18:1, and 20:1 and higher 18:2 and 18:3 relative to Col‐0. Overall, nitrogen supply reduced total oil similarly in both genotypes, whereas *cruabc* showed a reproducible shift toward more unsaturated and less elongated acyl chains across nitrogen levels.

### Seed storage proteins buffer oxidative stress under drought and high nitrogen conditions

Because mutants lacking major seed storage proteins show altered glutathione pools (Bagaza et al., 2024; Bagaza et al., 2025), we quantified reduced (GSH) and oxidized (GSSG) glutathione and calculated the GSH:GSSG ratio as a redox state indicator across genotypes and environments.

#### Water treatments

**Figure 5:**
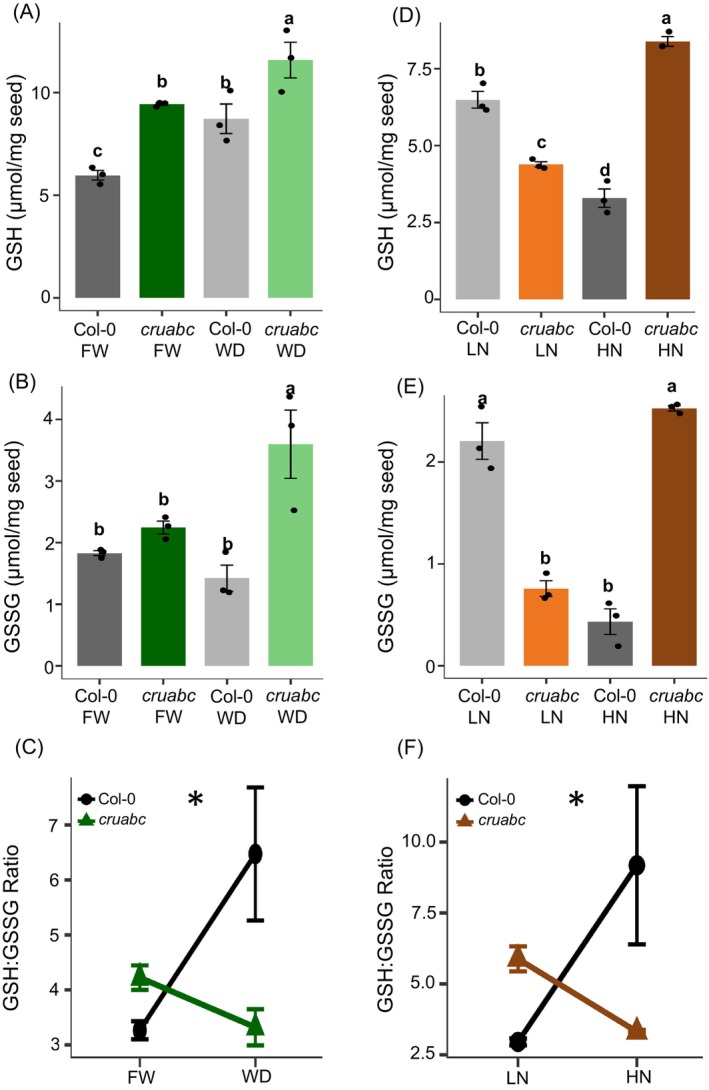
Water deficit and excess nitrogen increased glutathione levels in the dry seed of *cruabc*. (A, B) reduced glutathione (GSH) under water‐deficit and nitrogen treatments, respectively. (C, D) oxidized glutathione (GSSG) under water‐deficit and nitrogen treatments, respectively. (E, F) The ratio between GSH and GSSG under water‐deficit and nitrogen treatments, respectively. Error bars represent standard errors (*n* = 3). A Duncan's multiple range test was used to compare the genotypes, with different lowercase letters indicating significant differences at the 5% level. Points represent individual biological replicates. Asterisk indicates a significant genotype × treatment interaction determined by two‐way ANOVA (*P* < 0.05; ns, not significant).

Under FW, *cruabc* already contains more GSH than Col‐0. Under WD, GSH increased in both genotypes; however, the relative increase was greater in Col‐0 than in *cruabc* (Figure 5A). In contrast, GSSG increased only in *cruabc* under WD and did not significantly change in Col‐0 (Figure 5B). Hence, the GSH:GSSG ratio indicates that *cruabc* is overall more reduced at FW compared to Col‐0. Under WD, however, the GSH:GSSG ratio diverged, making *cruabc* more oxidized at WD conditions relative to Col‐0 (Figure 5C).

#### Nitrogen treatments

Nitrogen availability altered glutathione pools in opposite directions between genotypes (Figure 5D, E). In Col‐0, both GSH and GSSG were higher under LN than HN, consistent with elevated oxidative pressure under nitrogen limitation. In contrast, *cruabc* showed lower GSH and GSSG under LN and higher levels under HN (Figure 5D,E). The GSH:GSSG ratio exhibited a significant genotype × nitrogen interaction, with Col‐0 showing higher ratios than *cruabc* under HN, whereas under LN, the genotypes were more similar (Figure 5F). This pattern suggests reduced oxidative stress in *cruabc* when nitrogen is limiting.

### Genotype‐ and environment‐dependent proteome remodeling maintains global seed proteome homeostasis

To evaluate broader proteome changes in mature seeds, we performed comparative proteomics analysis on dry seeds from Col‐0 and *cruabc* grown under FW versus WD and LN versus HN. Across all treatments, we reliably identified, quantified, and normalized 3231 seed proteins (Data S4A). Differential abundance was assessed for *cruabc* versus Col‐0 within each treatment (FW, WD, LN, HN), and proteins were called differentially expressed (DEPs) when the *cruabc*/Col‐0 ratio was ≥1.2 or ≤0.8 (≥ 20% change) with FDR <0.05. To separate genotype and environmental effects on the proteome, we also compared *cruabc* and Col‐0 within each treatment and contrasted water or nitrogen regimes within each genotype, allowing identification of genotype‐, environment‐, and G × E‐dependent protein responses. Volcano and MA plots (Figure S4) summarize these patterns, revealing symmetric up‐ and downregulation and broader dynamic range under stress. These measurements represent the steady‐state proteome composition of mature seeds and thus capture the integrated outcome of developmental and stress‐induced processes occurring during seed filling.

#### Water treatments

**Figure 6:**
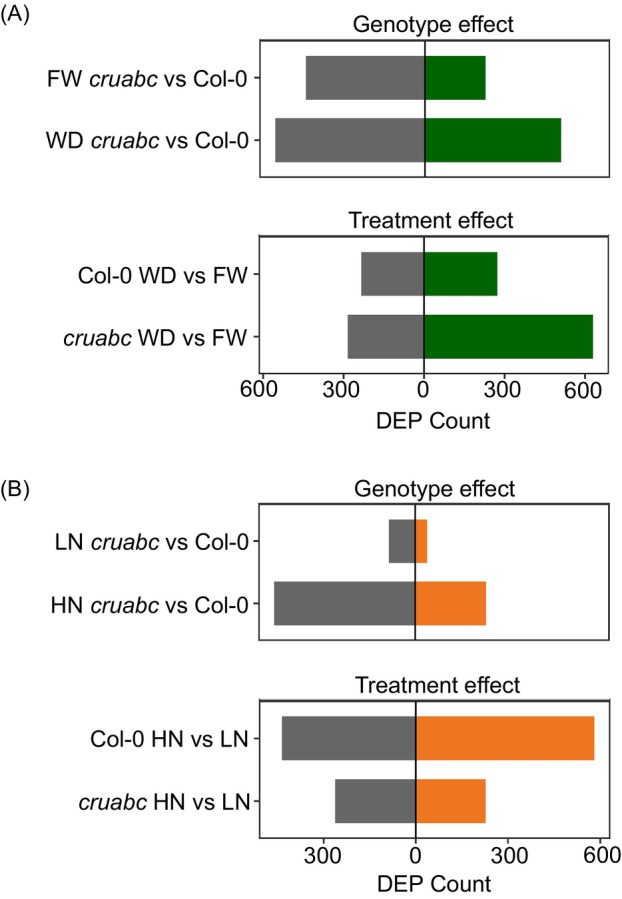
Overview of proteomic responses in Col‐0 and *cruabc* seeds under nitrogen and water treatments. (A) Numbers of differentially expressed proteins (DEPs) for the genotype effect (*cruabc* versus Col‐0 within the same treatment) and the treatment effect (within the same genotype) under water conditions (WD versus FW). (B) Corresponding DEP counts for the same comparisons under nitrogen conditions (HN versus LN). DEP significance was determined at FDR ≤0.05 and fold‐change ≥1.2 or ≤0.8.

A substantial number of proteins differed between genotypes under both watering conditions (Figure 6A; Data S4B). Under FW, 669 DEPs (226 up‐ and 443 downregulated) were detected; this number nearly doubled under WD to 1064 DEPs (507 upregulated and 557 downregulated), indicating enhanced drought‐associated proteomic divergence between the two genotypes.

*Within‐genotype treatment effect* revealed 507 WD–FW DEPs in Col‐0 (274 upregulated, 233 downregulated), whereas *cruabc* exhibited a broader drought response with 914 DEPs (630 upregulated, 284 downregulated), supporting a broader drought response in the mutant background.

#### Nitrogen treatments

Genotype differences were modest under LN, with only 124 DEPs (39 upregulated, 85 downregulated), but increased sharply under HN to 689 DEPs (231 upregulated, 458 downregulated), suggesting that nitrogen availability accentuates genotype‐specific proteome remodeling (Figure 6B; Data S4C). *Treatment effects* were stronger in Col‐0 than in *cruabc*: between HN and LN, Col‐0 had 1016 DEPs (581 upregulated, 435 downregulated), whereas *cruabc* displayed 489 DEPs (227 upregulated, 262 downregulated). This indicates that the wild type mounts a broader nitrogen‐responsive proteome shift, while CRUs' loss constrains nitrogen‐dependent protein turnover or synthesis in mature seeds.

### Drought triggers stronger and more diverse proteome remodeling in *cruabc* than in Col‐0

**Figure 7:**
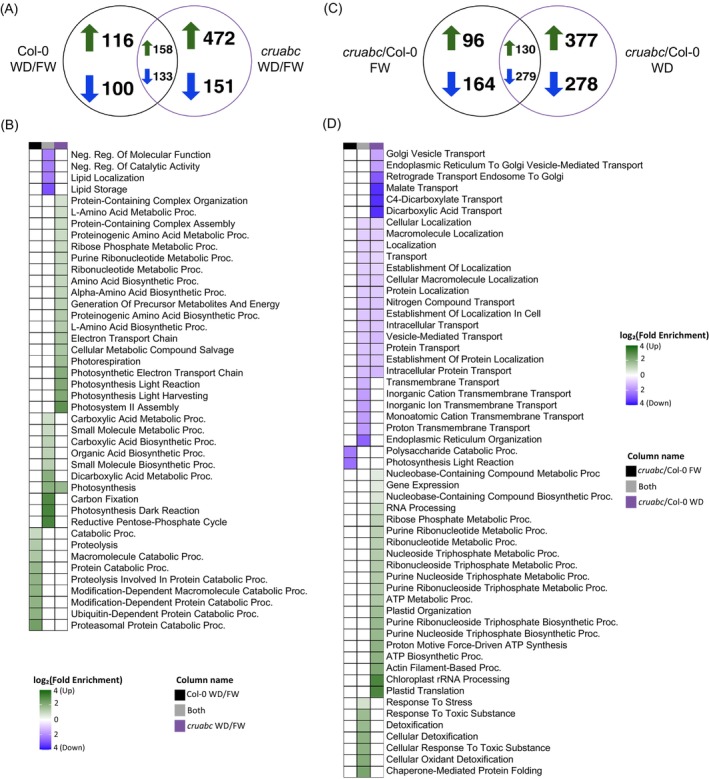
Proteomic responses of Col‐0 and *cruabc* seeds under drought conditions. (A) Venn diagram showing the overlap of increased and decreased differentially expressed DEPs for the treatment effect (WD versus FW within each genotype). (B) GO enrichment heat map (biological process category) corresponding to the same treatment effect comparison. (C) Venn diagram showing the overlap of increased and decreased DEPs for the genotype effect (*cruabc* versus Col‐0 within the same water condition). (D) GO enrichment heat map (biological process category) corresponding to the same genotype effects. Color scale represents log_2_(Fold Enrichment), where green indicates enrichment among upregulated DEPs and blue indicates enrichment among downregulated DEPs.

To characterize drought‐responsive proteome shifts, we first identified DEPs between WD and FW conditions within each genotype and compared shared and genotype‐specific responses (Figure 7A). The *cruabc* mutant exhibited substantially more drought‐induced changes than Col‐0; among WD‐increased proteins, *cruabc* had 472 unique DEPs compared with 116 in Col‐0, with 158 shared DEPs. For WD‐decreased proteins, *cruabc* had 151 unique DEPs and Col‐0 had 100, with 133 shared DEPs. These patterns indicate that *cruabc* undergoes substantially broader proteome reprogramming under drought.

Gene Ontology (GO) enrichment analysis showed that drought elicited a relatively limited proteomic response in Col‐0, with catabolic and proteostasis pathways enriched among WD‐increased proteins and no significant BP enrichment among WD‐decreased proteins (Figure 7B; Figure S5B). Molecular function enrichment among WD‐decreased proteins unique to Col‐0, which were enriched for thioredoxin‐dependent peroxiredoxin activity, indicated a mild redox adjustment under water stress (Figure S5B). In contrast, *cruabc* exhibited broader enrichment among WD‐increased proteins, including amino acid metabolism, electron transport, energy and photosynthesis‐related processes, and cellular compound salvage pathways, reflecting a far more comprehensive metabolic adjustment under WD (Figure 7B; Figure S5B). No significant enrichment was found among WD‐decreased proteins in *cruabc*. Proteins increased in both genotypes were enriched for photosynthesis, carbon fixation, carboxylic acid biosynthesis and metabolism, and the reductive pentose phosphate cycle, indicating a conserved metabolic core activated under drought stress (Figure 7B). Shared‐up proteins were enriched for MF GO terms for RNA and mRNA binding, pointing to enhanced translational or post‐transcriptional regulation under WD (Figure S5B). Conversely, the shared‐down DEPs were enriched in lipid localization and storage and negative regulation of molecular and catalytic activity (Figure 7B). Overall, Col‐0 exhibits a relatively narrow, proteostasis‐centered drought response, whereas *cruabc* activates a broader metabolic, energetic, and stress‐responsive remodeling program, consistent with a pre‐existing stress‐like state. Full GO enrichment data are available in Data S5A.

Because our previous work showed that the *cruabc* mutant strongly affects translational and redox machinery (Bagaza et al., 2024), we next tested whether these systems also exhibit differences under contrasting environmental conditions. To assess drought effects on translation, we extracted DEPs associated with ribosomal proteins and translation eIFs/EFs from WD–FW comparisons within each genotype (Figure S6A,B). The *cruabc* mutant exhibited substantially more increases in translation‐related proteins under drought (31 versus 8 in Col‐0), with 13 shared between genotypes. For WD‐decreased translation‐related proteins, *cruabc* again showed more unique decreases (15 versus 1 in Col‐0), with four shared (Figure S6A,B). Together, these results indicate that drought activates translational machinery in both genotypes but far more extensively in *cruabc*, consistent with its heightened proteome plasticity.

Analysis of ROS‐ and antioxidant‐associated DEPs revealed a substantially stronger drought‐induced redox response in *cruabc* (Figure S6C,D). Among WD‐increased proteins, *cruabc* showed 29 genotype‐specific increases compared with eight in Col‐0, with nine shared. *cruabc* also exhibited broader WD decreases in ROS‐related proteins. The log_2_ (FC) heat maps highlight strong upregulation of multiple ROS‐scavenging enzymes and redox regulators in *cruabc*, consistent with enhanced antioxidant remodeling under drought stress.

### Genotype‐specific proteome differences within each environment reveal core CRU functions and drought‐driven divergence

To assess the genotype effects across water treatments, we compared *cruabc* and Col‐0 within FW and WD conditions to identify proteins that differ between genotypes in a treatment‐specific or shared manner (Figure 7C). This approach distinguishes constitutive CRU‐dependent processes from drought‐specific adaptive responses. In this analysis across water treatments, *cruabc* versus Col‐0 contrasts in FW and WD were compared to determine shared and treatment‐specific genotype effects. Among proteins with higher abundance in *cruabc*, 377 were water treatment‐specific compared to 96 in Col‐0, with 130 shared between FW and WD. Conversely, among lower abundance proteins, 278 were water treatment‐specific in *cruabc* versus 164 in Col‐0, with 279 shared (Figure 7C).

*Under FW*, *cruabc* and Col‐0 displayed no significant enrichment among proteins higher in *cruabc* in the dry seed. However, proteins lower in *cruabc* under FW were enriched for polysaccharide catabolic processes and photosynthesis light reactions, suggesting reduced investment in carbohydrate turnover and plastid light‐associated activity under non‐stress conditions (Figure 7D). No significant molecular function (MF) enrichment was detected among either increased or decreased proteins, consistent with relatively few functional shifts between genotypes in the absence of environmental stress (Figure S5D).

*Under WD*, *cruabc*‐specific WD‐upregulated proteins were enriched in plastid translation, chloroplast rRNA processing, ATP biosynthetic processes, and purine nucleoside and ribonucleoside triphosphate biosynthesis (Figure 7D). MF enrichment among these proteins included ADP binding, proton channel activity, and RNA/mRNA binding, pointing to enhanced plastid gene expression and energy‐coupled remodeling under drought (Figure S5D). In contrast, *cruabc*‐specific WD‐downregulated proteins were enriched for dicarboxylic acid transport, malate transport, retrograde endosome‐to‐Golgi trafficking, and macromolecule localization, suggesting selective downregulation of transport‐related and trafficking pathways (Figure 7D). Proteins consistently more abundant in both genotypes under WD (shared‐up DEPs) were enriched for response to stress, response to toxic substances, cellular detoxification, and oxidant detoxification, signifying a conserved drought‐protective module active in both genetic backgrounds (Figure 7D). Corresponding MF terms included acetyl‐CoA carboxylase, peroxidase, antioxidant, and translation regulator activities, alongside copper ion binding, consistent with enhanced redox buffering and stress adaptation mechanisms (Figure S5D). Conversely, shared‐down proteins were enriched for endoplasmic reticulum organization, proton and cation transmembrane transport, and vesicle‐mediated transport, while MF terms included epoxide hydrolase activity, ether hydrolase activity, FAD binding, and transporter activity. Full enrichment data for genotype effects under FW and WD are provided in Data S5B.

### Nitrogen supply triggers renewed proteome remodeling in *cruabc*, whereas low nitrogen reflects a baseline rebalanced state

**Figure 8:**
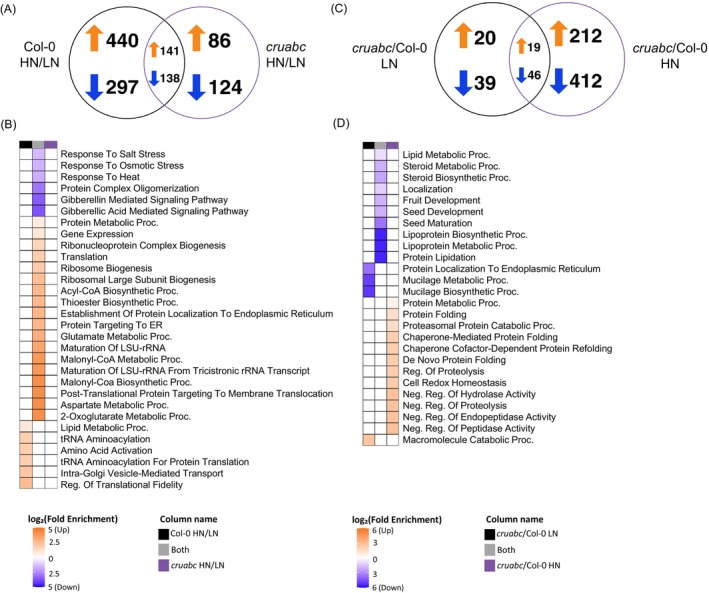
Proteomic responses of Col‐0 and *cruabc* seeds under various nitrogen treatments. (A) Venn diagram showing the overlap of increased and decreased DEPs for the treatment effect (HN versus LN within each genotype). (B) GO enrichment heat map (biological process category) corresponding to the same treatment effect comparison. No significant enrichment was found for *cruabc* HN/LN DEPs. (C) Venn diagram showing the overlap of increased and decreased DEPs for the genotype effect (*cruabc* versus Col‐0 within the same nitrogen treatment). (D) GO enrichment heat map (biological process category) corresponding to the same genotype effects. Color scale represents log_2_(Fold Enrichment), where orange indicates enrichment among upregulated DEPs and blue indicates enrichment among downregulated DEPs.

To assess nitrogen‐dependent proteome shifts, we first compared HN versus LN within each genotype (Figure 8A). Col‐0 undergoes a far broader shift than *cruabc*: 440 HN‐increased and 297 HN‐decreased proteins are unique to Col‐0, whereas *cruabc* shows 86 HN‐increased and 124 HN‐decreased proteins unique to the mutant; a core set is shared by both genotypes (141 increased, 138 decreased). Thus, HN elicits extensive proteomic reconfiguration in Col‐0, while *cruabc* exhibits a more constrained response, suggesting that nitrogen availability differentially modulates proteome remodeling in the mutant.

Among the unique Col‐0 HN‐increased proteins, BP enrichment was dominated by lipid metabolic processes, tRNA aminoacylation, amino acid activation, and regulation of translational fidelity, highlighting strong induction of protein synthesis and membrane remodeling under nitrogen excess (Figure 8B). The corresponding molecular function (MF) terms emphasized mRNA 5′‐UTR binding, aminoacyl‐tRNA ligase activity, and catalytic functions acting on tRNAs, consistent with enhanced translational capacity (Figure S7B). In contrast, *cruabc* HN‐increased proteins showed no significant BP or MF enrichment, reflecting a more constrained and selective proteomic response to nitrogen excess (Figure 8B; Figure S7B).

For the unique HN‐decreased proteins, Col‐0 displayed MF enrichment for thioredoxin peroxidase activity, proteasome binding, and antioxidant activity, suggesting a shift away from redox maintenance toward anabolic functions. The *cruabc* mutant again exhibited distinct signatures: while BP terms were not significantly enriched, MF enrichment included β‐N‐acetylhexosaminidase and carotenoid dioxygenase activities, structural constituents of chromatin, and hydrolases acting on glycosyl bonds, indicating selective metabolic downregulation (Figure 8B; Figure S7B).

The shared upregulated set between both genotypes was enriched for canonical nitrogen‐responsive anabolic categories, including ribosome biogenesis, translation, acetyl‐CoA and malonyl‐CoA metabolism, glutamate and aspartate metabolism, and post‐translational protein targeting to membranes (Figure 8B), with MF terms dominated by structural constituents of ribosomes, mRNA/RNA binding, and L‐aspartate:2‐oxoglutarate aminotransferase activity (Figure S7B). Conversely, the shared downregulated proteins were enriched for stress and hormone‐responsive pathways such as responses to salt, osmotic, and heat stress and gibberellin‐mediated signaling, indicating a coordinated suppression of environmental response functions under nitrogen excess. Together, these profiles show that both genotypes activate core translational and biosynthetic responses to HN, but Col‐0 displays broader anabolic upregulation, whereas *cruabc* remains comparatively buffered with limited translational reactivation and weaker suppression of stress and antioxidant processes.

To evaluate how nitrogen availability impacts the translational machinery, we extracted all DEPs associated with ribosomal proteins and translation eIFs/EFs from the treatment‐effect comparisons (HN/LN) for each genotype (Figure S8A,B). There were 38 Col‐0‐unique HN‐increased proteins, 42 shared HN‐increased proteins, and eight *cruabc*‐unique HN‐increased proteins. Decreases are sparse: 12 Col‐0‐unique HN‐decreased, one *cruabc*‐unique HN‐decreased, and no shared decreases (Figure S8A). Thus, nitrogen excess broadly elevates translational components in both genotypes but far more extensively in Col‐0. The log_2_(FC) heat map illustrates the composition of these protein abundance shifts (Figure S8B).

To assess how nitrogen availability influences antioxidants and ROS‐related proteins, we examined redox‐associated DEPs under HN/LN within each genotype (Figure S8C,D). Col‐0 had 16 uniquely increased and 24 uniquely decreased proteins, while *cruabc* displayed four uniquely increased and 14 uniquely decreased proteins. Only four ROS‐related proteins were commonly upregulated and nine commonly downregulated between the two genotypes (Figure S8C).

### Nitrogen availability amplifies proteome differences between genotypes

To assess nitrogen‐dependent genotype effects, we compared *cruabc* and Col‐0 within low‐ and high‐nitrogen conditions (Figure 8C). Under HN, *cruabc* showed extensive divergence from Col‐0, with 212 proteins upregulated and 412 downregulated, whereas under LN only modest genotype differences were observed (20 upregulated and 39 downregulated proteins, with 19 and 46 shared, respectively). These patterns indicate that proteome differences are strongly amplified under HN, whereas under LN *cruabc* and Col‐0 maintain highly similar proteomic profiles, consistent with LN stress being associated with limited proteome reprogramming and reduced genotype‐dependent divergence.

*Under LN, cruabc* displayed minimal enrichment among upregulated proteins, with only macromolecule catabolic processes emerging within the biological process (BP) category and no significant molecular function (MF) terms detected (Figure 8D; Figure S7D). Conversely, LN‐decreased proteins in *cruabc* were enriched for mucilage biosynthetic and metabolic processes as well as protein localization to the endoplasmic reticulum, accompanied by MF enrichment for *cis*‐regulatory region sequence‐specific DNA binding (Figure 8D; Figure S7D).

*Under HN*, *cruabc* HN‐increased proteins were strongly enriched for negative regulation of peptidase and endopeptidase activity, proteolysis, and hydrolase activity, alongside de novo protein folding and chaperone‐mediated folding, indicating enhanced proteostasis and chaperone engagement under nutrient excess (Figure 8D). Corresponding MF terms included copper‐ion binding, antioxidant activity, protein‐folding chaperone function, and peptidase inhibitor activity, collectively reflecting an upregulation of stress‐mitigating and protein quality control machinery (Figure S7D). In contrast, HN‐decreased proteins showed no significant BP or MF enrichment.

For shared genotype effects across LN and HN, no significant enrichment was detected among shared upregulated proteins, whereas shared downregulated proteins were enriched for protein lipidation, lipoprotein metabolic process, seed maturation, seed development, steroid and lipid metabolism, with MF terms centered on nutrient reservoir, peroxygenase, lipase, and glucosidase activities (Figure 8D; Figure S7D). These patterns indicate that *cruabc* consistently downregulates seed storage and lipid‐associated processes, irrespective of nitrogen level, while nitrogen excess preferentially induces chaperone‐ and antioxidant‐linked mechanisms that distinguish its proteomic response from Col‐0.

### Core translational and redox remodeling underlies proteome rebalancing across environments

To identify translation‐related proteins constitutively associated with proteomic rebalancing, we compiled all eukaryotic initiation factors (eIFs), elongation factors (EFs), and ribosomal proteins (RPs) consistently altered in *cruabc* relative to Col‐0 across at least three of the four experimental conditions (Table 1). This analysis revealed a stable translational signature marked by coordinated induction and repression of distinct translational components. A core set of translational components was consistently upregulated across all conditions, including the elongation factors EF1δ and EF1β; several translation initiation factors (eIF2β, eIF3G, eIF3I, eIF3J); and the large‐subunit ribosomal proteins uL29w and uL29z. In contrast, a set of ribosomal proteins putatively associated with SSP translation was consistently repressed across all treatments, including eL6x, eL6z, uL1y, uL1z, uS2z, uS3z, and uS8z. This dichotomy indicates a long‐term rewiring of the seed translational landscape rather than environment‐specific regulation.

**Table 1:** Translational components consistently altered in the *cruabc* mutant relative to Col‐0 across multiple treatments

Gene	Symbol	Name	Regulation
AT1G30230	EF1δ	Elongation factor 1 δ	Up
AT5G20920	eIF2β	Eukaryotic Translation Initiation Factor 2 subunit β	Up
AT3G11400	eIF3G	Eukaryotic Translation Initiation Factor 3 subunit G	Up
AT2G46280	eIF3I	Eukaryotic Translation Initiation Factor 3 subunit I	Up
AT5G37475	eIF3J	Eukaryotic Translation Initiation Factor 3 subunit J	Up
AT5G19510	EF1B	Putative elongation factor 1B α‐subunit	Up
AT5G02610	uL29w	Ribosomal Protein uL29w	Up
AT3G09500	uL29z	Ribosomal Protein uL29z	Up
AT3G02200	eIF3M	Eukaryotic Translation Initiation Factor 3 subunit M	Down
AT1G74050	eL6x	Ribosomal Protein eL6x	Down
AT1G18540	eL6z	Ribosomal Protein eL6z	Down
AT2G27530	uL1y	Ribosomal Protein uL1y	Down
AT1G08360	uL1z	Ribosomal Protein uL1z	Down
AT5G23740	uS17x*	Ribosomal Protein uS17x	Down
AT1G72370	uS2z	Ribosomal Protein uS2z	Down
AT2G31610	uS3z	Ribosomal Protein uS3z	Down
AT1G07770	uS8z	Ribosomal Protein uS8z	Down

*Note*: Shown are eukaryotic translation initiation factors (eIFs), elongation factors (EFs), and ribosomal proteins (RPs) that were consistently up‐ or downregulated under at least three conditions—high nitrogen (HN), low nitrogen (LN), full water (FW), and water deficit (WD). Proteins marked with an asterisk (*) were consistently regulated in all four conditions.

To identify redox components constitutively altered during proteome rebalancing, we compiled antioxidant and ROS‐associated proteins consistently regulated across at least three environmental treatments (Table 2). Proteins consistently upregulated in *cruabc* included multiple key mitochondrial antioxidants—superoxide dismutase (MSD1), glutaredoxin C2 (GRXC2), peroxiredoxins PRXIIB and PRXIIF, and glutathione peroxidase GPX6—as well as dehydroascorbate reductase (DHAR1) and succinate semialdehyde dehydrogenase (ALDH5F1). These sustained increases suggest a strengthened mitochondrial ROS‐scavenging and NADPH‐linked redox buffering system, likely supporting maintenance of metabolic flux under variable water and nitrogen conditions. Additionally, *cruabc* maintained elevated levels of methionine‐sulfoxide reductase B2 (MSRB2), a chloroplastic enzyme that protects translational and photosynthetic proteins from oxidative damage. In contrast, aldehyde oxidase 4 (AAO4), long‐chain acyl‐CoA synthetase 6 (LACS6), galactinol synthase 2 (GOLS2), berberine bridge enzyme‐like protein 24 (K9L2.18), tocopherol cyclase (VTE1), and the autophagy‐related protein ATG18A were consistently downregulated across all treatments, indicating selective attenuation of lipid redox and secondary antioxidant pathways.

**Table 2:** Reactive oxygen species (ROS)‐related proteins consistently altered in the *cruabc* mutant relative to Col‐0 across multiple treatments

Gene	Symbol	Name	Regulation
AT1G79440	ALDH5F1	Succinate‐semialdehyde dehydrogenase, mitochondrial	Up
AT1G19570	DHAR1	Glutathione S‐transferase DHAR1, mitochondrial	Up
AT3G10920	MSD1	Superoxide dismutase [Mn] 1, mitochondrial	Up
AT1G65980	PRXIIB	Peroxiredoxin‐2B	Up
AT3G06050	PRXIIF	Peroxiredoxin‐2F, mitochondrial	Up
AT3G53990	F5K20_290	Adenine nucleotide alpha hydrolases‐like superfamily protein.	Up
AT4G11600	GPX6	Probable phospholipid hydroperoxide glutathione peroxidase 6, mitochondrial	Up
AT5G40370	GRXC2	Glutaredoxin‐C2	Up
AT4G21860	MSRB2	Peptide methionine sulfoxide reductase B2, chloroplastic	Up
AT1G04580	AAO4	Aldehyde oxidase 4	Down
AT3G05970	LACS6	Long chain acyl‐CoA synthetase 6, peroxisomal	Down
AT1G56600	GOLS2	Galactinol synthase 2	Down
AT5G44380	K9L2.18	Berberine bridge enzyme‐like 24.	Down
AT4G32770	VTE1	Tocopherol cyclase, chloroplastic	Down
AT3G62770	ATG18A*	Autophagy‐related protein 18a	Down

*Note*: Listed are antioxidant enzymes and redox‐associated proteins that were consistently up‐ or downregulated under at least three conditions—high nitrogen (HN), low nitrogen (LN), full water (FW), and water deficit (WD). Proteins marked with an asterisk (*) were significantly regulated in all four treatments.

Together, these coordinated translational and redox shifts define a balanced, energy‐efficient antioxidant architecture that stabilizes cellular homeostasis and supports proteome maintenance during storage protein rebalancing.

## DISCUSSION

### Robust seed proteome rebalancing under genetic and environmental perturbations

Seed proteome rebalancing, defined as the capacity of developing seeds to compensate for large genetic perturbations such as the elimination of SSPs while maintaining overall amino acid composition, is conserved across species yet remains mechanistically largely unresolved. Our previous multi‐omics work in *Arabidopsis thaliana* has shown that rebalancing involves modulation of the translational machinery, including changes in ribosomal protein (RP) composition, implicating ribosome heterogeneity in compensatory protein synthesis (Bagaza et al., 2024). Here, we extend this framework by examining how rebalancing persists when SSP loss (*cruabc*; elimination of the three major 12S cruciferins) is combined with environmental stress. Remarkably, across contrasting treatments—(i) severe drought (water deficit, WD) during seed filling and (ii) variable nitrogen (N) availability—final seed amino acid composition in *cruabc* remained broadly comparable to the wild type (WT), despite distinct metabolic and proteomic adjustments in each genotype (Figures 1 and 2; Tables S2–S5). These findings indicate that proteome rebalancing represents a stable homeostatic endpoint that can be reset in response to environmental changes. However, the metabolic routes and energetic costs required to reach this endpoint differ substantially between genotypes, revealing how SSP loss reshapes stress adaptation during seed maturation and desiccation.

An important consideration is that all analyses were performed on mature dry seeds, which represent the terminal, desiccated state of the seed proteome. Consequently, differences in protein abundance reflect not real‐time regulatory activity, but the integrated outcomes of developmental and stress‐induced processes operating during seed filling, together with the selective stability and persistence of proteins through maturation. In our previous work, we have shown that proteome rebalancing is not an instantaneous response limited to one time point but rather a developmentally integrated process, in which transient regulatory changes are consolidated into a stable proteome composition (Bagaza et al., 2024). During seed maturation, storage proteins are actively synthesized and deposited, while their turnover is minimal, resulting in a highly stable protein reservoir (Müntz et al., 2001). Accordingly, proteins that accumulate in mature seeds are those that are either strongly produced during late seed filling or inherently stable, whereas proteins with more transient roles, particularly those involved in active translation or redox signaling, are likely to be underrepresented due to turnover prior to desiccation. Thus, processes such as translational control and redox remodeling, which are expected to be most dynamic during late‐stage seed development when stress is imposed, may not be fully captured at the level of steady‐state abundance in dry seeds. Instead, the mature seed proteome reflects the subset of proteins that have been preferentially synthesized, stabilized, and retained through maturation.

Importantly, because stress treatments were applied during seed filling, the mature seed captures the cumulative outcome of these responses, even if peak regulatory dynamics occurred earlier. The proteomic, translational, and redox‐related changes described here should therefore be interpreted as stabilized endpoints of proteome rebalancing, integrating both differential synthesis during development and selective persistence into the mature seed. In this way, our analysis provides insight into the functionally relevant outputs of proteome rebalancing, complementing studies that focus on dynamic regulation during seed development.

### Drought stress increases the energetic and redox cost of rebalancing

Under drought, Col‐0 plants displayed a conservative compensation strategy: yield and seed size declined, but total carbon and nitrogen per seed were largely maintained (Figure 1). Proteomics enrichment analysis revealed a classic drought signature, with increased representation of catabolic and proteolytic processes and decreased enrichment of lipid metabolism‐related terms, aligning with the observed reduction in total oil content and oleic acid content under WD (Figure 4A,C). These trends align with prior reports in maize and soybean showing drought‐induced reductions in oil content and altered fatty acid composition (Ali et al., 2010; Ali et al., 2012; Dornbos Jr & Mullen, 1992). Notably, dry seeds of both genotypes were enriched in photosynthesis‐related proteins and components of the reductive pentose phosphate pathway (Figure 7B), suggesting localized CO_2_ refixation and enhanced redox buffering during seed maturation under limited assimilate supply. Similar embryo‐localized photosynthetic activity has been reported in *Brassica napus* and *Camelina sativa*, where it improves carbon‐use efficiency and supports energy balance under stress (Eastmond et al., 1996) (Carey et al., 2020).

In *cruabc*, proteome rebalancing persisted under drought but appeared to incur higher energetic costs and require broader proteomic reprogramming, as reflected by the greater number of up‐ and downregulated DEPs in the treatment‐effect comparisons (Figure 7A). Free amino acids (FAAs), particularly proline and valine (Figures 2B, 3B; Figure S3C), increased sharply, consistent with their roles in osmotic adjustment, redox buffering, and metabolic reprogramming for stress endurance (Gipson et al., 2017; Hayat et al., 2012; Verslues & Sharma, 2010). Moreover, both bound and free forms of threonine and valine exhibited significant genotype × treatment interactions, amino acid accumulation in *cruabc* under WD (Figure 3A,B), suggesting elevated metabolic flux through amino acid biosynthesis. The enrichment of GO categories such as amino acid biosynthesis, energy‐yielding metabolic processes, ribose phosphate metabolism, electron transport, and photorespiration suggests that *cruabc* experiences heightened energetic demand (Figure 7B).

While both genotypes showed reduced levels of total oil content under drought (Figure 4A), *cruabc* exhibited a stronger decrease in oleic acid and a concomitant increase in α‐linolenic acid (Figure 4C), suggesting genotype‐specific remodeling of fatty acid desaturation. This shift may relate to previously reported differences in lipid body organization in *cruabc* (Bagaza et al., 2024), potentially reflecting altered lipid packaging and turnover or oxidative constraints. Redox profiling further underscored the cost of rebalancing under drought. Total glutathione (GSH) increased in both genotypes under drought conditions, but the accumulation was strongest in *cruabc* (Figure 5A). While Col‐0 maintained stable GSSG levels under drought, *cruabc* exhibited a significant rise in oxidized glutathione (GSSG; Figure 5B), resulting in a reduced GSH:GSSG ratio under drought (Figure 5C). Thus, although *cruabc* seeds are more reduced under control conditions (FW), they experience greater redox imbalance and oxidative stress under water limitation, likely reflecting higher metabolic and energetic demands associated with sustaining proteome rebalancing and stress responses.

Together, these data support that SSPs are not inert storage reserves but have key metabolic roles in seed maturation and provide redox‐stabilizing protective sinks. Their absence forces compensatory re‐routing of carbon and nitrogen through energetically costly pathways, potentially reducing physiological resilience under drought. This has direct implications for SSP‐targeted biofortification strategies, where reduced storage protein synthesis—such as in maize *opaque2*—can incur stress sensitivity or yield penalties under suboptimal environments (Gupta et al., 2009; Salamini et al., 1970).

### Nitrogen availability is a major determinant of proteome rebalancing

In Col‐0 plants, increasing external N input increased single seed weight and yield and SSP accumulation (Figure 1E,F; Figure S1), responses widely reported in crop seeds (Bonfanti et al., 2025; Ou et al., 2025). Increasing nitrogen availability reduced total seed oil content (Figure 4B). In Col‐0, the shift from low to HN decreased gondoic (20:1) and stearic (18:0) acids while increasing oleic (18:1) and α‐linolenic (18:3) acids (Figure 4D), indicating nitrogen‐dependent remodeling of fatty acid composition, consistent with reports in other oilseed crops (Zapletalová et al., 2021; Zhu et al., 2023). HN markedly increased seed nitrogen content in Col‐0 seeds on both a per‐seed and per‐mass basis, while carbon content increased slightly per seed (Figure 1G,H). The shift from LN to HN was accompanied by a strong increase in TPBAAs (Figure 2C) and a modest rise in TFAAs (Figure 2D).

Despite these differences, overall seed amino acid composition in *cruabc* remained similar to Col‐0 across nitrogen regimes (Figure 2C,F), with the exception of a marked increase in the FAA pool under HN (Figure 2D). However, sink performance diverged strongly: *cruabc* showed higher yield under LN but reduced yield under HN (Figure 1E). This reversal suggests that SSPs provide an efficient carbon and nitrogen sink under nitrogen sufficiency, whereas their synthesis becomes energetically costly under limitation. Consistent with this view, *cruabc* rebalancing is lower in magnitude under LN, as evidenced by the small number of genotype‐dependent proteomic differences under LN (Figures 6B and 8C), whereas divergence increases under HN.

Proteomic analysis of Col‐0 seeds showed that HN enriched GO terms related to translation, including tRNA aminoacylation, amino acid activation, and translational fidelity (Figure 8B), consistent with enhanced translational capacity under nitrogen sufficiency via amino acid–TOR signaling (Busche et al., 2021; Tulin et al., 2021). In contrast, these translational enrichments were largely absent in *cruabc*, despite comparable TPBAA levels under HN (Figure 2C). This suggests that increased nitrogen availability does not proportionally enhance translational investment in the mutant, likely due to altered sink capacity. HN also suppressed stress‐response pathways and GA‐related processes in both genotypes (Figure 8B), consistent with a shift toward ABA‐mediated maturation (Ali et al., 2022).

Genotype × environment interaction analysis revealed significant correlations for free glutamine (Gln), aspartate (Asp), and asparagine (Asn) between low‐ and high‐nitrogen treatments in both genotypes, though the magnitude was substantially higher in *cruabc* (Figure 3D). These nitrogen‐rich amino acids are central intermediates in nitrogen assimilation and signaling. Notably, Gln was recently identified as the principal exogenous nutrient activator of the target of rapamycin (TOR) pathway in developing pea embryos (O'Leary et al., 2025). The evolutionarily conserved TOR kinase integrates nitrogen and carbon nutrient signals to coordinate growth, translation, and anabolic metabolism while suppressing catabolic processes such as autophagy, thereby maintaining cellular homeostasis (Dobrenel et al., 2016). Thus, under nitrogen sufficiency, elevated Gln levels are expected to stimulate TOR activity.

Redox responses diverged markedly between genotypes. While mature seeds are typically in an oxidized redox state to maintain dormancy (Gerna et al., 2017; Morscher et al., 2015), the Col‐0 seeds under HN exhibited an unusually high GSH:GSSG ratio (Figure 5F), indicative of sustained metabolic activity. In contrast, *cruabc* became more oxidized under HN (Figure 5F), consistent with progression toward maturation. These contrasting redox responses suggest that nitrogen enrichment prolongs metabolic activity in the WT, whereas *cruabc*, despite its altered proteome, follows a more typical redox trajectory toward maturation.

Under LN, genotype effects (*cruabc* versus Col‐0) revealed upregulation of catabolic processes and downregulation of mucilage biosynthesis and ER‐associated protein localization, indicating a shift toward resource mobilization rather than storage (Figure 8D). Reduced mucilage‐related processes mirror phenotypes of raptor1b mutants with impaired TOR signaling, linking SSP loss to altered nutrient signaling (Salem et al., 2017). Across both nitrogen regimes, *cruabc* consistently showed repression of lipid metabolism, seed maturation, and fruit development terms, reflecting broadly dampened reserve accumulation programs independent of nitrogen availability (Figure 8D). Although total oil content declined from LN to HN as in Col‐0 (Figure 4B), *cruabc* exhibited a persistently more unsaturated oil profile, enriched in linoleic and α‐linolenic acids (Figure 4D), increasing susceptibility to lipid peroxidation (Li et al., 2022). Consistent with this, *cruabc* seeds displayed a more oxidized redox state under HN despite elevated glutathione, indicating that loss of SSPs imposes coupled energetic and oxidative burdens during seed maturation and storage.

### Rebalancing signature: Selective translational reprogramming and redox control

Across all treatments, rebalancing was associated with coordinated remodeling of translational and redox components (Figures S6 and S8). A conserved translational module emerged, characterized by consistent upregulation of initiation and elongation factors (eIF2β, eIF3G/I/J, EF1δ, EF1B) and remodeling of ribosomal proteins: uL29 paralogs (located at the peptide‐exit tunnel) were elevated, whereas RPs associated with SSP translation (uS2, uS3, uS8, uS17, uL1, and eL6) were repressed (Figures S6A,B and S8A,B; Table 1). Given uL29's role at the peptide exit tunnel in coordinating nascent‐chain folding and quality control (Contreras‐Martinez et al., 2012; Kramer et al., 2009), this pattern supports ribosome specialization and selective translation rather than increased bulk protein synthesis. The selective accumulation of eIF3G and eIF3I suggests reconfiguration of the eIF3 complex toward translation of stress‐ or resource‐responsive mRNAs, consistent with reports in animals and yeast demonstrating subunit‐specific translational specificity (Blazie et al., 2021) (Stanciu et al., 2021).

Among the consistently repressed translational proteins, eIF3M (AT3G02200) is a 5′TOP mRNA regulated by TOR–LARP1 signaling (Scarpin et al., 2022), directly linking translational remodeling to nutrient‐responsive control in *cruabc*. Studies in yeast and human cells indicate that eIF3M defines a specialized eIF3 complex required for translational competence and context‐dependent mRNA selection (Goh et al., 2011; Zhou et al., 2005). We propose that SSP loss decouples canonical storage protein‐primed translation, redirecting ribosomal capacity toward compensatory metabolic and stress‐response programs.

Redox proteome profiling revealed a conserved antioxidant module operating in parallel (Table 2). ROS‐related proteins consistently regulated across treatments revealed a coherent antioxidant response dominated by mitochondrial and chloroplastic components (Table 2). Upregulation of MSD1, GRXC2, PRXIIB/F, GPX6, DHAR1, and ALDH5F1 defines a robust coordinated ROS‐scavenging and NADPH‐recycling module. Conversely, downregulation of AAO4, LACS6, GOLS2, VTE1, and ATG18A highlights a selective suppression of lipid‐derived and secondary antioxidant pathways. This shift is consistent with *cruabc* seeds adopting a more energetically conservative, steady‐state antioxidant strategy, which may contribute to proteome stability under sustained rebalancing.

## CONCLUSION

This study identifies three core principles governing seed proteome rebalancing. First, SSP loss shifts seeds toward a nitrogen‐limited–like metabolic and proteomic state associated with constrained proteome remodeling and altered nitrogen allocation. Second, SSPs function as metabolically efficient nitrogen sinks, conferring advantages under nitrogen sufficiency but imposing costs under limitation, highlighting nitrogen availability as a critical parameter for effective seed biofortification strategies. Third, SSPs contribute to redox buffering; their absence heightens oxidative pressure despite compensatory antioxidant reinforcement. Together, these findings position SSP‐linked translational control and redox feedback as central integrators of carbon, nitrogen, and energy balance in seeds, providing a conceptual framework for engineering nutritionally enhanced yet resilient crops.

## EXPERIMENTAL PROCEDURES

### Plant material and growth conditions

Arabidopsis thaliana triple mutant lines (*cruabc*) were kindly provided by Dr. Dwayne D. Hegedus (Agriculture and Agri‐Food Canada, Saskatoon, Saskatchewan, S7N 0X2, Canada). The *cruabc* line is a homozygous triple mutant generated by crossing three T‐DNA insertion lines targeting *CRUA*, *CRUB*, and *CRUC*, as previously described (Withana‐Gamage et al., 2013). Seeds from *cruabc* and wild‐type Columbia‐0 (Col‐0) were sown and grown under controlled conditions in a growth chamber set to 24°C/22°C (day/night) with a long‐day photoperiod (16 h light/8 h dark). Plants were well watered throughout vegetative growth (FW). For drought treatments (water deficit, WD), water was withheld beginning at the onset of flowering (approximately 4 weeks before harvest). For nitrogen treatments, seeds were grown under low nitrogen (LN) and high nitrogen (HN) conditions. For the low‐nitrogen (LN) treatment, plants were initially irrigated with a modified Bausenwein nutrient solution (Thornton & Bausenwein, 2000) prepared in deionized water, adjusted to pH 5.6, and using Fe‐EDTA in place of FeCl_3_ as the iron source, and were subsequently watered with deionized water only until maturity. For the HN treatment, plants were grown using the modified Bausenwein medium, and beginning at bolting, they were supplemented weekly with ammonium nitrate solution. At maturity, seeds were harvested and air‐dried under ambient conditions prior to further analyses. All analyses were conducted on freshly harvested mature (dry) seeds and therefore reflect the accumulated proteome and metabolite composition following seed development rather than dynamic processes occurring during earlier developmental stages.

### Seed weight

Average single seed weight was determined by weighing approximately 1000 seeds (*n* = 4 biological replicates) from both *cruabc* and Col‐0 plants grown under different treatments. The total weight was divided by the seed count to calculate the mean single seed weight (mg).

### Seed yield

Seeds were harvested throughout the seed setting period from all treatments (*n* = 5) to prevent seed loss. The final seed yield was determined by combining all the harvests from a single pot.

### Seed moisture content

Seed moisture content was measured following the method described by Baud et al. (2002). Approximately 10 mg of seed from three biological replicates per treatment (*n* = 3) was weighed (W1), then dried at 50°C for 48 hours and reweighed (W2). Seed moisture was calculated using the formula 100 × (W1 − W2)/W2, where W1 is the weight before drying and W2 is the weight after drying. Moreover, dry weight (DW) was determined accordingly.

### Nitrogen and carbon analyses

Total nitrogen and carbon were quantified using an ECS 4010 CHNS‐O elemental combustion system analyzer (NC TECHNOLOGIES). Approximately 8 mg of dry seed tissue was used per sample, with three biological replicates (*n* = 3) per treatment across two genotypes.

### Oil/lipid analysis

Oil analysis was performed using ~15 mg seeds from Col‐0 and *cruabc* (*n* = 3) based on Folch et al. (1957). Briefly, extraction was performed using a 5% chloroform (v/v) in methanol, and the analysis was performed using a GC with a flame ionization detector on a polar column–like DB23 (30 m by 0.25 mm id., 0.25 μm film; J&W Scientific, Folsom, CA).

### SDS‐PAGE and protein visualization

Total proteins were extracted from ~20 mg of dry seed tissue using extraction buffer containing 200 mM Tris‐HCl (pH 8.0), 1% SDS, 20% glycerol, and 1% β‐mercaptoethanol. Samples were bead‐beaten for 5 min, heated at 95 °C for 5 min, and centrifuged at 3700*g* for 30 min at 4°C. Equal volumes of the clarified supernatants were loaded onto 4%–12% gradient SDS–polyacrylamide gels and electrophoresed at 120 V. Gels were stained with Coomassie Brilliant Blue R‐250 and destained in 40% methanol/10% acetic acid.

### Amino acid analysis

Protein‐bound amino acids (PBAAs) were extracted as described by Yobi and Angelovici (2018). Briefly, ~3 mg of dry seeds from five biological replicates (*n* = 5) was hydrolyzed in 6 N HCl at 110°C for 24 h. The resulting hydrolysates were analyzed using an ultra‐performance liquid chromatography‐tandem mass spectrometer (UPLC‐MS/MS; Waters Corporation, Milford, MA). This method enabled quantification of 16 amino acids, representing 17 due to the conversion of asparagine (Asn) and glutamine (Gln) to aspartate (Asp) and glutamate (Glu), respectively. These are reported as combined totals: Asx (Asn + Asp) and Glx (Gln + Glu). Free amino acids (FAAs) were extracted as described in Ansaf et al. (2024). Dry seeds were analyzed without further processing (*n* = 4).

### Glutathione analysis

Glutathione quantification was performed using approximately 20 mg of dry seeds (*n* = 3) and the glutathione assay kit (Cayman Chemical, Ann Arbor, MI), following the protocol described in Bagaza et al. (2024). Reduced glutathione (GSH) levels were measured after treatment with metaphosphoric acid (MPA), while oxidized glutathione (GSSG) was assessed following derivatization with 2‐vinylpyridine. Samples were incubated at room temperature for 60 min, followed by the addition of the assay cocktail. After shaking in the dark, absorbance was measured at 405 nm. GSH and GSSG concentrations were determined by comparison to standard curves prepared from known GSH and GSSG standards. Final concentrations were calculated by applying the appropriate dilution factors and normalizing to the sample weight, as detailed in Bagaza et al. (2024).

### Proteome analysis

Protein extraction was carried out using a Tris‐HCl‐buffered phenol and SDS‐based protocol, followed by precipitation with 0.1 M ammonium acetate in methanol, as described in Bagaza et al. (2024). Approximately 5 mg of dry seed tissue was used per sample. Proteins were centrifuged, washed with 80% acetone, dried, and resuspended in urea buffer. Quantification was performed using the Pierce 660 nm Protein Assay. Equal protein amounts (25 μg per sample) were digested with trypsin in two rounds. Peptides were purified using C18 tips, lyophilized, and resuspended in solvent prior to analysis. Samples were analyzed on a Bruker timsTOF Pro2 mass spectrometer coupled with an Evosep One system using a 44‐minute gradient method. Initial proteome profiling was performed using a Bruker nanoElute system connected to a timsTOF Pro, operated in positive‐ion data‐independent acquisition (DIA) PASEF mode across an m/z range of 400–1200 and an ion mobility (IM) range of 0.57–1.47 1/K₀. DIA data were processed in Spectronaut v18.1 using a previously generated spectral library based on Arabidopsis seeds and the TAIR11 protein database. Default Pulsar search settings were used, with the following parameters: enzyme = trypsin/P, peptide length = 7–52 amino acids, up to two missed cleavages, carbamidomethylation of cysteine as a fixed modification, and oxidation (M) and acetylation (protein N terminus) as variable modifications. Identification thresholds were set at 1% FDR for PSM, peptide, and protein levels, and only protein group‐specific peptides were used (prototypicity filter enabled). Protein quantification was based on MaxLFQ using MS2 peak area, with cross‐run normalization enabled and no imputation applied. For differential analysis, proteins with fold‐change ratios between 0.8 and 1.2 (mutant versus control) were excluded. The remaining proteins were subjected to limma‐moderated *t*‐tests, and significance was determined after applying a 5% FDR correction using limma R package (Ritchie et al., 2015). Differentially abundant proteins (DEPs) were analyzed across eight key comparisons to assess genotype‐ and treatment‐specific responses. These included cruabc WD versus Col‐0 WD, cruabc FW versus Col‐0 FW, cruabc WD versus cruabc FW, and Col‐0 WD versus Col‐0 FW, as well as cruabc low nitrogen (LN) versus Col‐0 LN, cruabc high nitrogen (HN) versus Col‐0 HN, cruabc HN versus cruabc LN, and Col‐0 HN versus Col‐0 LN. The mass spectrometry proteomics data have been deposited to the ProteomeXchange Consortium via the PRIDE partner repository with the dataset identifier “PXD071067.”

### Bioinformatics analysis

GO enrichment analysis was conducted using ShinyGO v0.82 with Arabidopsis thaliana as the reference organism and an FDR threshold of 0.05 (Ge et al., 2020). The background for enrichment consisted of proteins reliably detected in our proteomics dataset. The “Remove redundancy” option was selected to eliminate similar pathways that share 95% of their genes and 50% of the words in their names, representing them with the pathway that has the highest significance. The top 20 GO enrichment terms from ShinyGO were used to construct enrichment heat maps, which were created in R using ggplot2 v3.4.4, dplyr v1.1.4, tidyr v1.3.0, and scales v1.3.0. Ribosomal proteins were retrieved from Scarpin et al. (2023). The list of Arabidopsis eukaryotic translation initiation factors was retrieved from GO molecular function (MF) GO:0003743. The list of Arabidopsis elongation factors was retrieved from GO MF GO:0003746. To generate a curated ROS‐related gene set for downstream analyses, we extracted all genes annotated under the following Gene Ontology (GO) biological process terms: GO:0072593 (reactive oxygen species metabolic process), GO:0000302 (response to reactive oxygen species), GO:0034614 (cellular response to reactive oxygen species), GO:0006749 (glutathione metabolic process), and GO:0006979 (response to oxidative stress). Heat maps were generated in R (v4.4.3) using the ComplexHeatmap package. DIA relative protein abundance intensities were averaged across replicates, *Z*‐scored by row, and visualized using hierarchical clustering (Euclidean distance, complete linkage) for rows while preserving the original column order (no column clustering).

## AUTHOR CONTRIBUTIONS

HA: Investigation, data curation, formal analysis, visualization, writing—original draft, writing—review and editing. CB, AY, TPM: Investigation, writing—review and editing. RA: Supervision, funding acquisition, conceptualization, resources, writing—original draft, writing—review and editing.

## FUNDING INFORMATION

This work was funded by the NSF‐IOS 1754201 grant and NSF‐PGR 2350447.

## CONFLICT OF INTEREST STATEMENT

The authors have no conflict of interest to declare.

## Supporting information


**Dataset S1.** Protein‐bound amino acid (PBAA) and free amino acid (FAA) data from *cruabc* and Col‐0 at full water (FW) and water‐deficit (WD) conditions. (A) PBAA amino acid levels (nmol/mg). (B) PBAA amino acid composition (%PBAA/TPBAA). (C) FAA amino acid levels (nmol/mg). (D) FAA composition (%FAA/TFAA). TPBAA, total of all measured PBAAs per treatment; TFAA, total of all measured FAAs per treatment.

**Dataset S2.** Protein‐bound amino acid (PBAA) and free amino acid (FAA) data from *cruabc* and Col‐0 at low nitrogen (LN) and high nitrogen (HN) conditions. (A) PBAA amino acid levels (nmol/mg). (B) PBAA amino acid composition (%PBAA/TPBAA). (C) FAA amino acid levels (nmol/mg). (D) FAA composition (%FAA/TFAA). TPBAA, total of all measured PBAAs per treatment; TFAA, total of all measured FAAs per treatment.

**Dataset S3.** Seed fatty acid composition and total oil content in *cruabc* and Col‐0 under four independent treatments: low nitrogen (LN), high nitrogen (HN), full water (FW), and water deficit (WD). (A) Raw fatty acid data. (B) Averaged replicates.

**Dataset S4.** DIA‐based proteomic analysis of dry seeds from *cruabc* and Col‐0 under four treatments: full water (FW), water deficit (WD), low nitrogen (LN), and high nitrogen (HN). (A) Normalized DIA intensity data for all quantified proteins. (B) Differentially abundant proteins (DEPs) identified in *cruabc* versus Col‐0 for each treatment, with significance thresholds of FDR <0.05 and fold change ≥1.2 or ≤0.8. (C) Treatment‐effect DEP tables for FW, WD, LN, and HN comparisons (FDR <0.05 and fold change ≥1.2 or ≤0.8.).

**Dataset S5.** Gene Ontology (GO) enrichment (biological process, molecular function, cellular component) generated with ShinyGO v0.82 using Arabidopsis TAIR10 and all reliably detected proteins from the DIA experiment as background. (A, B) GO enrichment for drought comparisons: (A) treatment effect (WD versus FW) and (B) genotype effect (cruabc versus Col‐0). (C, D) GO enrichment for nitrogen comparisons: (C) treatment effect (HN versus LN), and (D) genotype effect (cruabc versus Col‐0). For all panels, terms were filtered at FDR <0.05, redundancy was removed, and up to 20 non‐redundant terms per ontology are shown, ranked by fold enrichment after FDR filtering.

**Figure S1.** Representative total protein profiles of Col‐0 and *cruabc* seeds under drought and nitrogen treatments. Coomassie Brilliant Blue‐stained SDS‐PAGE gel showing total soluble proteins extracted from dry seeds of Col‐0 and *cruabc* under water‐deficit (WD), well‐watered (FW), low‐nitrogen (LN), and high‐nitrogen (HN) conditions. Each lane represents an independent biological replicate. Samples were loaded by equal extraction volume rather than normalized protein concentration, resulting in visible differences in band intensity that reflect inherent variation in total protein abundance among treatments. Lane M is the molecular weight marker. Brackets denote the 12S cruciferin α‐ and β‐chains as well as the 2S proteins.
**Figure S2.** Non‐significant genotype × environment interactions FAAs and PBAAs for water treatment. A and B show protein‐bound amino acids (PBAAs) and free amino acids (FAAs), respectively, that exhibited non‐significant genotype × treatment interaction effects (*p* > 0.05) from a two‐way ANOVA. Each point represents the mean ± SE (*n* = 4–5) for each genotype under well‐watered (FW) and water‐deficit (WD) conditions.
**Figure S3.** Non‐significant genotype × environment interactions FAAs and PBAAs for nitrogen treatments. (A, B) Protein‐bound amino acids (PBAAs) and free amino acids (FAAs), respectively, that exhibited non‐significant genotype × treatment interaction effects (*P* > 0.05) from a two‐way ANOVA. Each point represents the mean ± SE (*n* = 4–5) for each genotype under low nitrogen (LN) and high nitrogen (HN) treatments.
**Figure S4.** Differential protein abundance for genotype effect and treatment effect comparisons. (A–D) Volcano (left) and MA (right) plots for drought experiments. (A, B) Genotype effects between *cruabc* and Col‐0 under (A) full‐water (FW) and (B) water‐deficit (WD) conditions. (C, D) Treatment‐effect comparisons within each genotype: (C) Col‐0 (WD versus FW) and (D) *cruabc* (WD versus FW). (E–H) Volcano (left) and MA (right) plots for nitrogen experiments. (E, F) Genotype effects between *cruabc* and Col‐0 under (E) low‐nitrogen (LN) and (F) high‐nitrogen (HN) conditions. (G, H) Treatment‐effect comparisons within each genotype: (G) Col‐0 (HN versus LN) and (H) *cruabc* (HN versus LN).
**Figure S5.** Proteomic responses of Col‐0 and *cruabc* seeds under drought conditions. (A) Venn diagram showing the overlap of increased and decreased DEPs for the treatment effect (WD versus FW within each genotype). (B) GO enrichment heat map (molecular function category) corresponding to the same treatment effect comparison. (C) Venn diagram showing the overlap of increased and decreased DEPs for the genotype effect (*cruabc* versus Col‐0 within the same water condition). (D) GO enrichment heat map (molecular function category) corresponding to the same genotype effects. Color scale represents log_2_(Fold Enrichment), where green indicates enrichment among increased DEPs and blue indicates enrichment among decreased DEPs.
**Figure S6.** Translational and ROS‐related protein responses to drought. (A)Venn diagram showing overlap of translational DEPs between Col‐0 and *cruabc* for the WD/FW comparison. (B) Heat map of log_2_ fold changes for translational DEPs. (C) Venn diagram showing overlap of ROS‐related DEPs between genotypes. (D)Heat map of log_2_ fold changes values for ROS‐related DEPs. Up‐ and downregulated proteins are shown in green and blue, respectively.
**Figure S7.** Proteomic responses of Col‐0 and *cruabc* seeds under various nitrogen treatments. (A) Venn diagram showing the overlap of increased and decreased DEPs for the treatment effect (HN vs LN within each genotype). (B) GO enrichment heat map (molecular function category) corresponding to the same treatment effect comparison. No significant enrichment was found for *cruabc* HN/LN DEPs. (C) Venn diagram showing the overlap of increased and decreased DEPs for the genotype effect (*cruabc* vs Col‐0 within the same nitrogen treatment). (D) GO enrichment heat map (molecular function category) corresponding to the same genotype effects. Color scale represents log_2_(Fold Enrichment), where orange indicates enrichment among upregulated DEPs and blue indicates enrichment among downregulated DEPs.
**Figure S8.** Translational and ROS‐related protein responses to nitrogen. (A) Venn diagram showing overlap of translational DEPs between Col‐0 and *cruabc* for the HN/LN comparison. (B) Heat map of log_2_ fold changes for translational DEPs. (C) Venn diagram showing overlap of ROS‐related DEPs between genotypes. (D) Heat map of log_2_FC values for ROS‐related DEPs. Up‐ and downregulated proteins are shown in orange and blue, respectively.

**Table S1.** Relative water content (RWC) of dry seeds harvested from *Arabidopsis* Col‐0 and *cruabc* plants subjected to various water and nitrogen treatments. The RWC was calculated as described in Material and Methods. A Duncan's multiple range test was used to compare the treatments, with same lowercase letters indicating no significant differences at the 5% level.
**Table S2.** Comparison of protein‐bound amino acids (PBAAs) and free amino acids (FAAs) between the *cruabc* mutant and the Col‐0 wild type under full‐water (FW) and water‐deficit (WD) conditions. (A) Average PBAA levels and corresponding fold changes (*cruabc*/Col‐0, *n* = 5). (B) Average FAA levels and corresponding fold changes (*cruabc*/Col‐0, *n* = 4). Significance was determined using a two‐sample *t*‐test; bold values indicate amino acids with significant increases or decreases at *P* < 0.05.
**Table S3.** Comparison of protein‐bound amino acid (PBAA) and free amino acid (FAA) composition between the *cruabc* mutant and the Col‐0 wild type under full‐water (FW) and water‐deficit (WD) conditions. (A) PBAA composition (%PBAA/total PBAA) and corresponding composition differences (%*cruabc* – %Col‐0). (B) FAA composition (%FAA/total FAA) and corresponding composition differences (%*cruabc* – %Col‐0). Significance was determined using a two‐sample *t*‐test; bold values indicate amino acids with significant increases or decreases at *P* < 0.05 (*n* = 5 for PBAAs, *n* = 4 for FAAs).
**Table S4.** Comparison of protein‐bound amino acids (PBAAs) and free amino acids (FAAs) between the *cruabc* mutant and the Col‐0 wild type under low‐nitrogen (LN) and high‐nitrogen (HN) conditions. (A) Average PBAA levels and corresponding fold changes (*cruabc*/Col‐0, *n* = 5). (B) Average FAA levels and corresponding fold changes (*cruabc*/Col‐0, *n* = 4). Significance was determined using a two‐sample *t*‐test; bold values indicate amino acids with significant increases or decreases at *P* < 0.05.
**Table S5.** Comparison of protein‐bound amino acid (PBAA) and free amino acid (FAA) composition between the *cruabc* mutant and the Col‐0 wild type under low‐nitrogen (LN) and high‐nitrogen (HN) conditions. (A) PBAA composition (%PBAA/total PBAA) and corresponding composition differences (% *cruabc* – %Col‐0). (B) FAA composition (%FAA/total FAA) and corresponding composition differences (% *cruabc* – %Col‐0). Significance was determined using a two‐sample *t*‐test; bold values indicate amino acids with significant increases or decreases at *P* < 0.05 (*n* = 5 for PBAAs, *n* = 4 for FAAs).

## Data Availability

The mass spectrometry proteomics data have been deposited to the ProteomeXchange Consortium via the PRIDE partner repository with the dataset identifier “PXD071067”.
