# Supplementary material for: Environmental stress reveals new insights regarding proteome rebalancing in Arabidopsis thaliana seeds
Source: Plant J. 2026 Apr 20;126(2):e70881. doi: 10.1111/tpj.70881 (PMC13094328; doi:10.1111/tpj.70881)
Supplement: Supplementary file 6 — Figure S1. Representative total protein profiles of Col‐0 and cruabc seeds under drought and nitrogen treatments. Coomassie Brilliant Blue‐stained SDS‐PAGE gel showing total soluble proteins extracted from dry seeds of Col‐0 and cruabc under water‐deficit (WD), well‐watered (FW), low‐nitrogen (LN), and high‐nitrogen (HN) conditions. Each lane represents an independent biological replicate. Samples were loaded by equal extraction volume rather than normalized protein concentration, resulting in visible differences in band intensity that reflect inherent variation in total protein abundance among treatments. Lane M is the molecular weight marker. Brackets denote the 12S cruciferin α‐ and β‐chains as well as the 2S proteins. Figure S2. Non‐significant genotype × environment interactions FAAs and PBAAs for water treatment. A and B show protein‐bound amino acids (PBAAs) and free amino acids (FAAs), respectively, that exhibited non‐significant genotype × treatment interaction effects (p > 0.05) from a two‐way ANOVA. Each point represents the mean ± SE (n = 4–5) for each genotype under well‐watered (FW) and water‐deficit (WD) conditions. Figure S3. Non‐significant genotype × environment interactions FAAs and PBAAs for nitrogen treatments. (A, B) Protein‐bound amino acids (PBAAs) and free amino acids (FAAs), respectively, that exhibited non‐significant genotype × treatment interaction effects (P > 0.05) from a two‐way ANOVA. Each point represents the mean ± SE (n = 4–5) for each genotype under low nitrogen (LN) and high nitrogen (HN) treatments. Figure S4. Differential protein abundance for genotype effect and treatment effect comparisons. (A–D) Volcano (left) and MA (right) plots for drought experiments. (A, B) Genotype effects between cruabc and Col‐0 under (A) full‐water (FW) and (B) water‐deficit (WD) conditions. (C, D) Treatment‐effect comparisons within each genotype: (C) Col‐0 (WD versus FW) and (D) cruabc (WD versus FW). (E–H) Volcano (left) and MA (right) plots for [file TPJ-126-0-s007.pptx]

## Slide 1
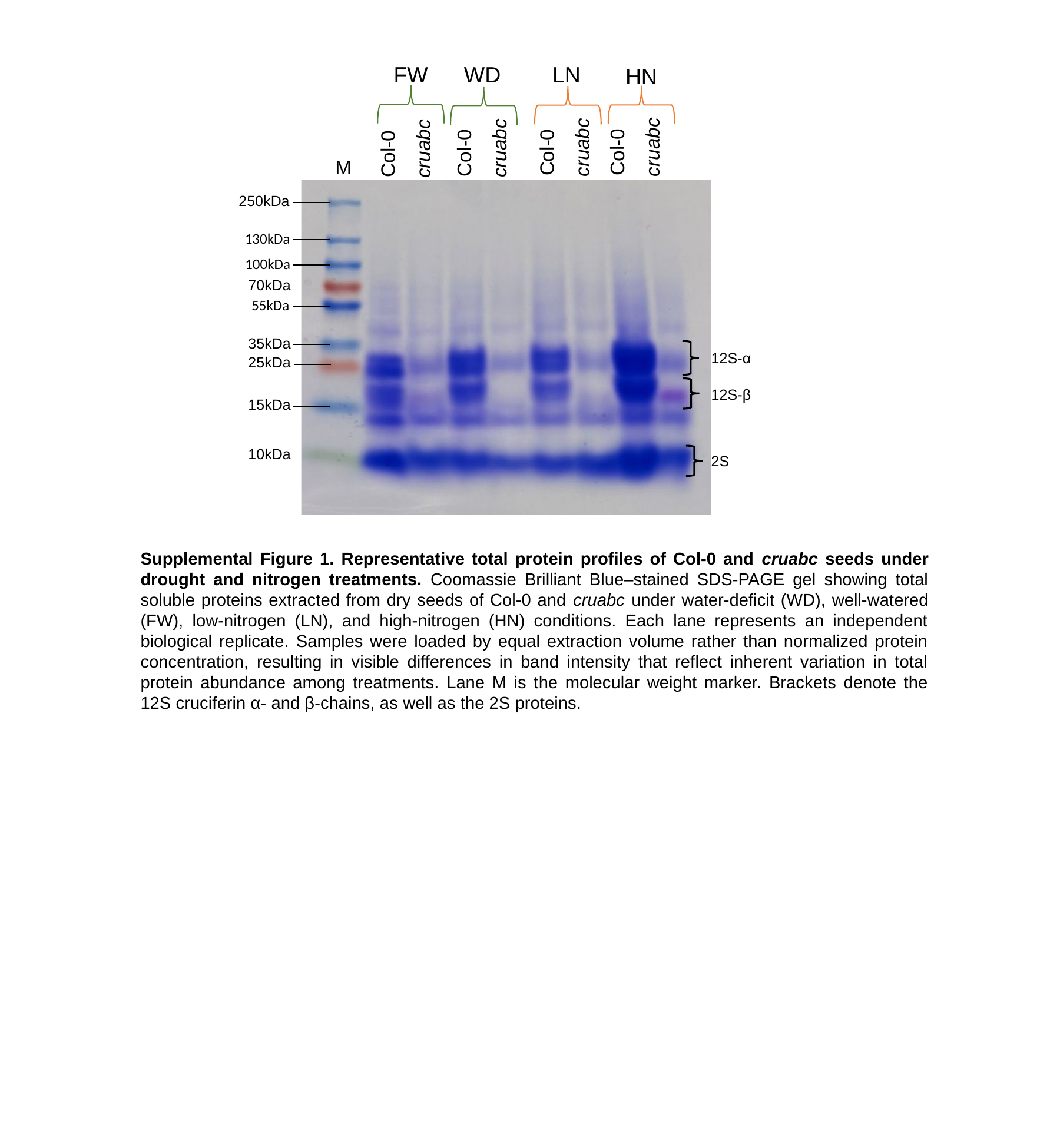

LN
WD
FW
HN
cruabc
cruabc
cruabc
cruabc
Col-0
Col-0
Col-0
Col-0
M
250kDa
70kDa
35kDa
25kDa
15kDa
10kDa
130kDa
100kDa
55kDa
12S-α
12S-β
2S
Supplemental Figure 1. Representative total protein profiles of Col-0 and cruabc seeds under drought and nitrogen treatments. Coomassie Brilliant Blue–stained SDS-PAGE gel showing total soluble proteins extracted from dry seeds of Col-0 and cruabc under water-deficit (WD), well-watered (FW), low-nitrogen (LN), and high-nitrogen (HN) conditions. Each lane represents an independent biological replicate. Samples were loaded by equal extraction volume rather than normalized protein concentration, resulting in visible differences in band intensity that reflect inherent variation in total protein abundance among treatments. Lane M is the molecular weight marker. Brackets denote the 12S cruciferin α- and β-chains, as well as the 2S proteins.

## Slide 2
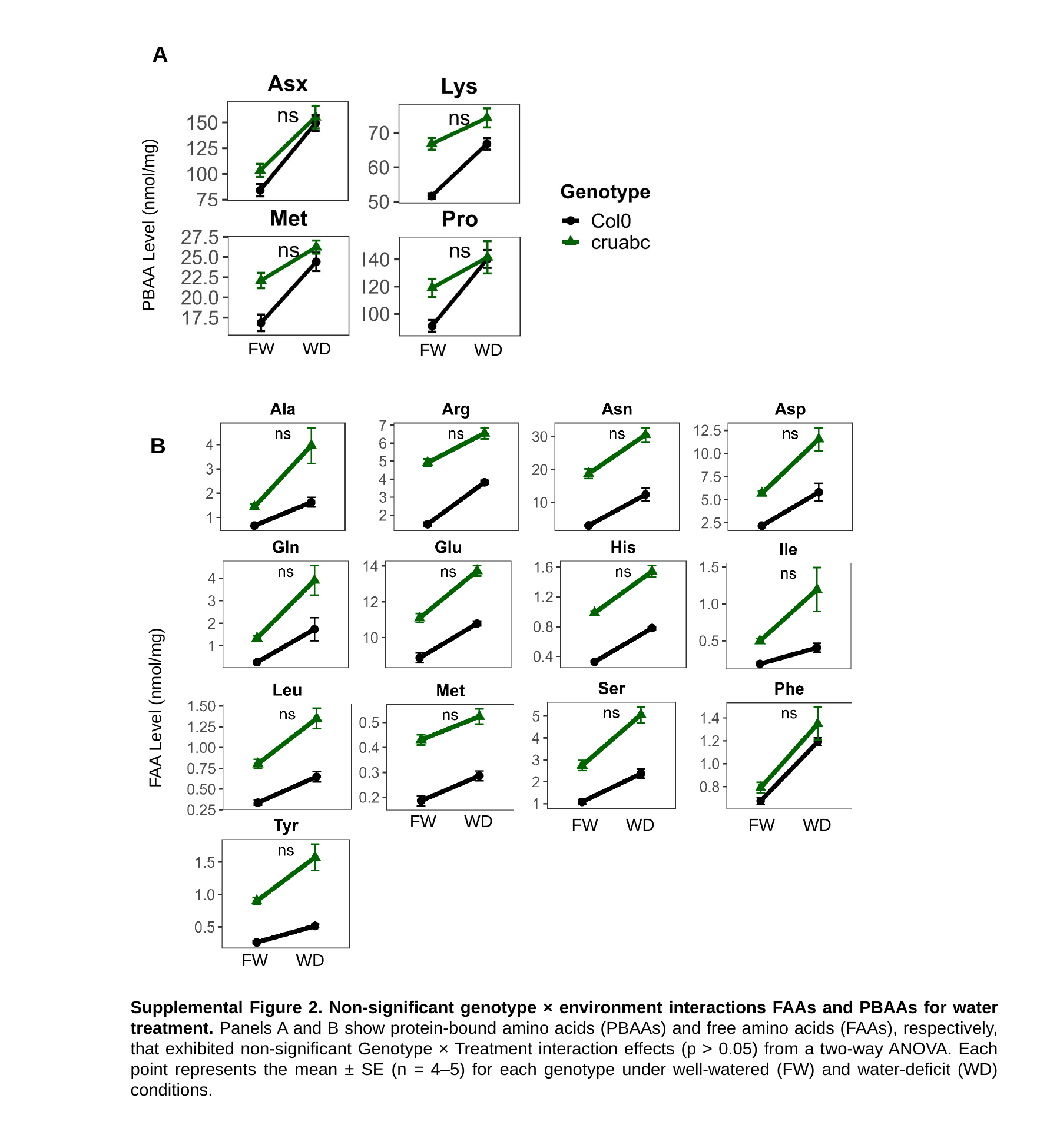

A
PBAA Level (nmol/mg)
FW
WD
FW
WD
B
FAA Level (nmol/mg)
FW
WD
FW
WD
FW
WD
FW
WD
Supplemental Figure 2. Non-significant genotype × environment interactions FAAs and PBAAs for water treatment. Panels A and B show protein-bound amino acids (PBAAs) and free amino acids (FAAs), respectively, that exhibited non-significant Genotype × Treatment interaction effects (p > 0.05) from a two-way ANOVA. Each point represents the mean ± SE (n = 4–5) for each genotype under well-watered (FW) and water-deficit (WD) conditions.

## Slide 3
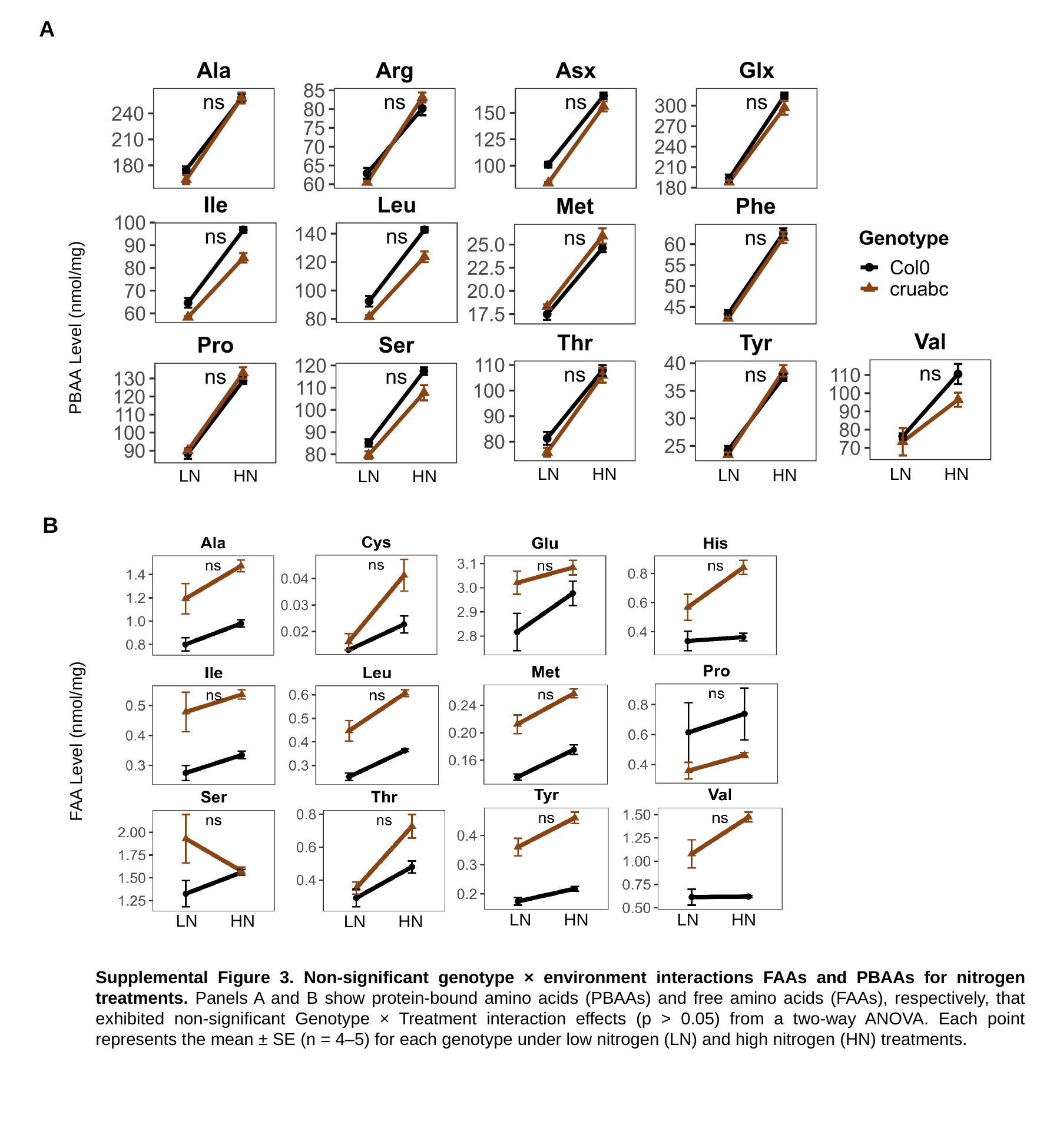

A
PBAA Level (nmol/mg)
LN
HN
LN
HN
LN
HN
LN
HN
LN
HN
B
FAA Level (nmol/mg)
LN
HN
LN
HN
LN
HN
LN
HN
Supplemental Figure 3. Non-significant genotype × environment interactions FAAs and PBAAs for nitrogen treatments. Panels A and B show protein-bound amino acids (PBAAs) and free amino acids (FAAs), respectively, that exhibited non-significant Genotype × Treatment interaction effects (p > 0.05) from a two-way ANOVA. Each point represents the mean ± SE (n = 4–5) for each genotype under low nitrogen (LN) and high nitrogen (HN) treatments.

## Slide 4
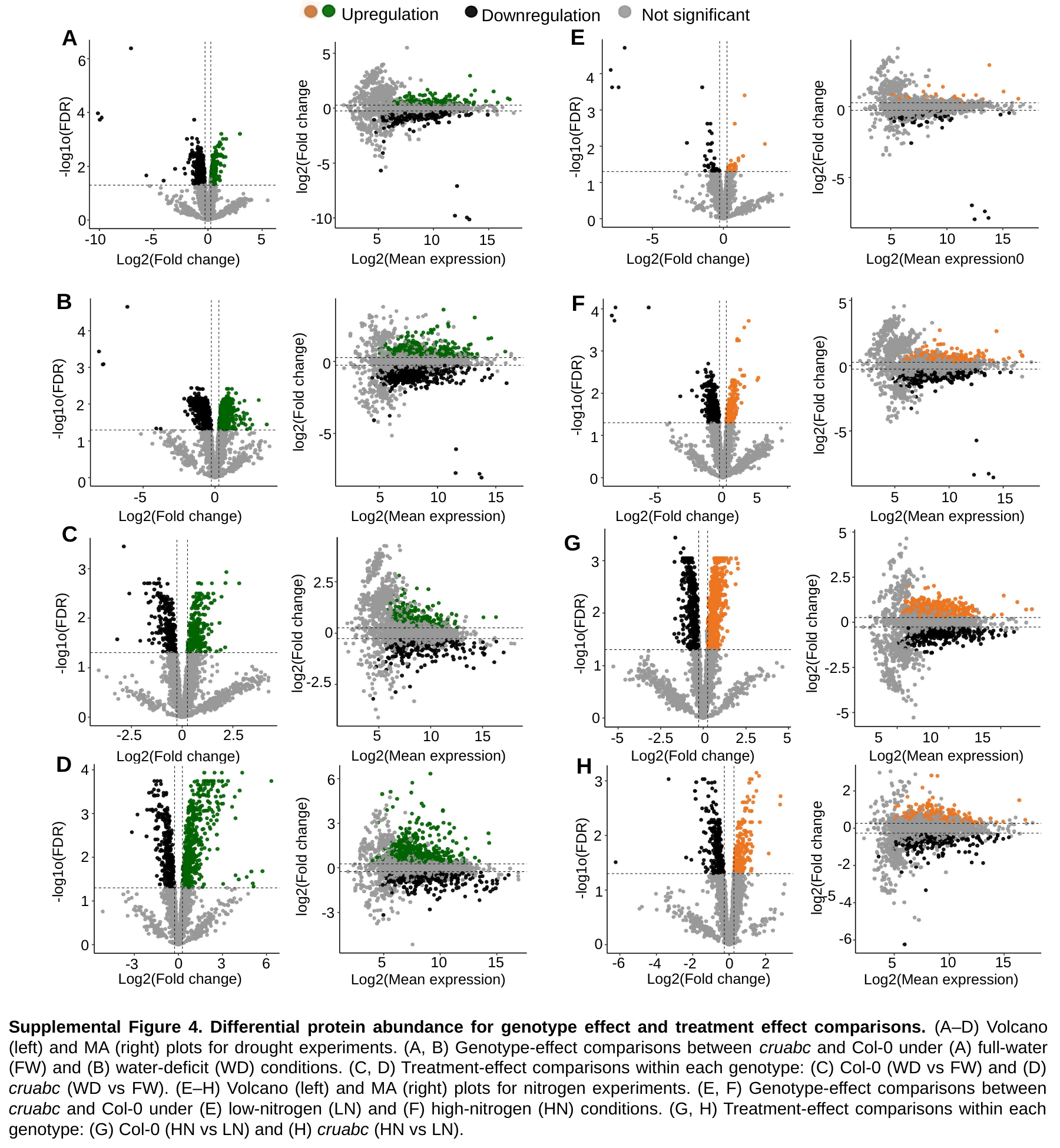

Upregulation
Not significant
Downregulation
4
3
-log1o(FDR)
2
1
0
0
-5
Log2(Fold change)
0
log2(Fold change
-5
15
10
5
Log2(Mean expression0
E
4
2
-log1o(FDR)
1
0
0
-5
F
3
5
5
0
log2(Fold change)
-5
10
5
15
Log2(Mean expression)
Log2(Fold change)
5
G
3
2
-log1o(FDR)
1
0
0
-5
Log2(Fold change)
0
log2(Fold change)
-5
5
15
10
Log2(Mean expression)
2.5
-2.5
5
-2.5
2.5
H
3
2
0
log2(Fold change
-5
10
15
5
Log2(Mean expression)
2
-log1o(FDR)
1
0
0
-2
2
Log2(Fold change)
-6
-4
A
6
4
-log1o(FDR)
2
0
0
-10
-5
5
Log2(Fold change)
4
3
-log1o(FDR)
2
1
0
0
-5
log2(Fold change)
Log2(Fold change)
B
C
3
2
-log1o(FDR)
1
0
-2.5
0
2.5
Log2(Fold change)
D
3
6
5
0
log2(Fold change
-5
-10
5
15
10
Log2(Mean expression)
0
-5
10
5
15
Log2(Mean expression)
2.5
0
log2(Fold change)
-2.5
5
15
10
Log2(Mean expression)
4
3
2
-log1o(FDR)
1
0
0
-3
log2(Fold change)
Log2(Fold change)
6
3
0
-3
10
5
15
Log2(Mean expression)
-2
-4
-6
Supplemental Figure 4. Differential protein abundance for genotype effect and treatment effect comparisons. (A–D) Volcano (left) and MA (right) plots for drought experiments. (A, B) Genotype-effect comparisons between cruabc and Col-0 under (A) full-water (FW) and (B) water-deficit (WD) conditions. (C, D) Treatment-effect comparisons within each genotype: (C) Col-0 (WD vs FW) and (D) cruabc (WD vs FW). (E–H) Volcano (left) and MA (right) plots for nitrogen experiments. (E, F) Genotype-effect comparisons between cruabc and Col-0 under (E) low-nitrogen (LN) and (F) high-nitrogen (HN) conditions. (G, H) Treatment-effect comparisons within each genotype: (G) Col-0 (HN vs LN) and (H) cruabc (HN vs LN).

## Slide 5
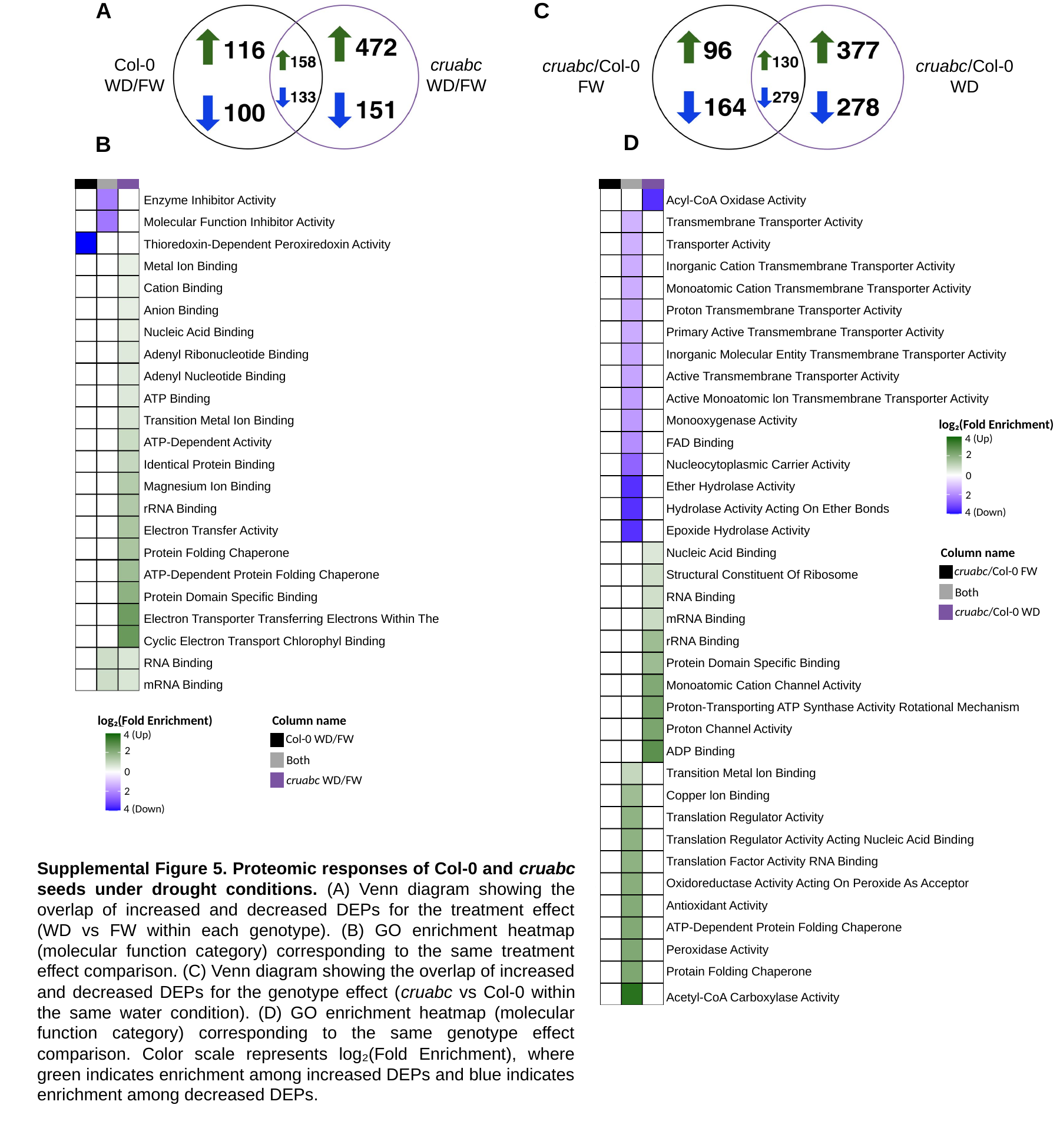

Col-0
WD/FW
cruabc
WD/FW
A
C
cruabc/Col-0
FW
cruabc/Col-0
WD
D
B
Acyl-CoA Oxidase Activity
Transmembrane Transporter Activity
Transporter Activity
Inorganic Cation Transmembrane Transporter Activity
Monoatomic Cation Transmembrane Transporter Activity
Proton Transmembrane Transporter Activity
Primary Active Transmembrane Transporter Activity
Inorganic Molecular Entity Transmembrane Transporter Activity
Active Transmembrane Transporter Activity
Active Monoatomic lon Transmembrane Transporter Activity
Monooxygenase Activity
FAD Binding
Nucleocytoplasmic Carrier Activity
Ether Hydrolase Activity
Hydrolase Activity Acting On Ether Bonds
Epoxide Hydrolase Activity
Nucleic Acid Binding
Structural Constituent Of Ribosome
RNA Binding
mRNA Binding
rRNA Binding
Protein Domain Specific Binding
Monoatomic Cation Channel Activity
Proton-Transporting ATP Synthase Activity Rotational Mechanism
Proton Channel Activity
ADP Binding
Transition Metal lon Binding
Copper lon Binding
Translation Regulator Activity
Translation Regulator Activity Acting Nucleic Acid Binding
Translation Factor Activity RNA Binding
Oxidoreductase Activity Acting On Peroxide As Acceptor
Antioxidant Activity
ATP-Dependent Protein Folding Chaperone
Peroxidase Activity
Protain Folding Chaperone
Acetyl-CoA Carboxylase Activity
log₂(Fold Enrichment)
4 (Up)
2
0
2
4 (Down)
Enzyme Inhibitor Activity
Molecular Function Inhibitor Activity
Thioredoxin-Dependent Peroxiredoxin Activity
Metal Ion Binding
Cation Binding
Anion Binding
Nucleic Acid Binding
Adenyl Ribonucleotide Binding
Adenyl Nucleotide Binding
ATP Binding
Transition Metal Ion Binding
ATP-Dependent Activity
Identical Protein Binding
Magnesium Ion Binding
rRNA Binding
Electron Transfer Activity
Protein Folding Chaperone
ATP-Dependent Protein Folding Chaperone
Protein Domain Specific Binding
Electron Transporter Transferring Electrons Within The Cyclic Electron Transport Chlorophyl Binding
RNA Binding
mRNA Binding
log₂(Fold Enrichment)
4 (Up)
2
0
2
4 (Down)
Column name
cruabc/Col-0 FW
Both
cruabc/Col-0 WD
Column name
Col-0 WD/FW
Both
cruabc WD/FW
Supplemental Figure 5. Proteomic responses of Col-0 and cruabc seeds under drought conditions. (A) Venn diagram showing the overlap of increased and decreased DEPs for the treatment effect (WD vs FW within each genotype). (B) GO enrichment heatmap (molecular function category) corresponding to the same treatment effect comparison. (C) Venn diagram showing the overlap of increased and decreased DEPs for the genotype effect (cruabc vs Col-0 within the same water condition). (D) GO enrichment heatmap (molecular function category) corresponding to the same genotype effect comparison. Color scale represents log₂(Fold Enrichment), where green indicates enrichment among increased DEPs and blue indicates enrichment among decreased DEPs.

## Slide 6
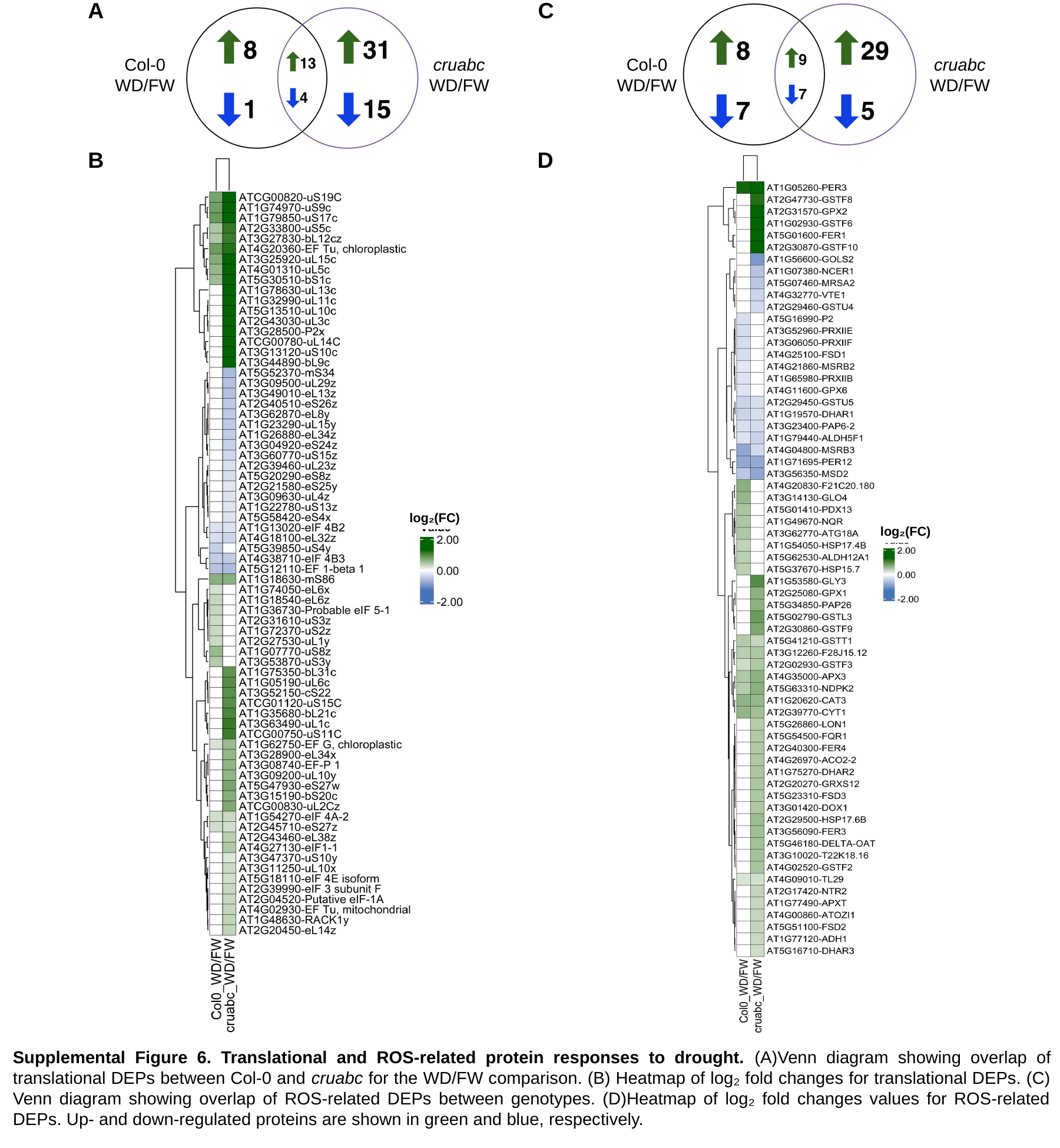

Col-0
WD/FW
cruabc
WD/FW
A
C
Col-0
WD/FW
cruabc
WD/FW
B
D
log₂(FC)
log₂(FC)
Supplemental Figure 6. Translational and ROS-related protein responses to drought. (A)Venn diagram showing overlap of translational DEPs between Col-0 and cruabc for the WD/FW comparison. (B) Heatmap of log₂ fold changes for translational DEPs. (C) Venn diagram showing overlap of ROS-related DEPs between genotypes. (D)Heatmap of log₂ fold changes values for ROS-related DEPs. Up- and down-regulated proteins are shown in green and blue, respectively.

## Slide 7
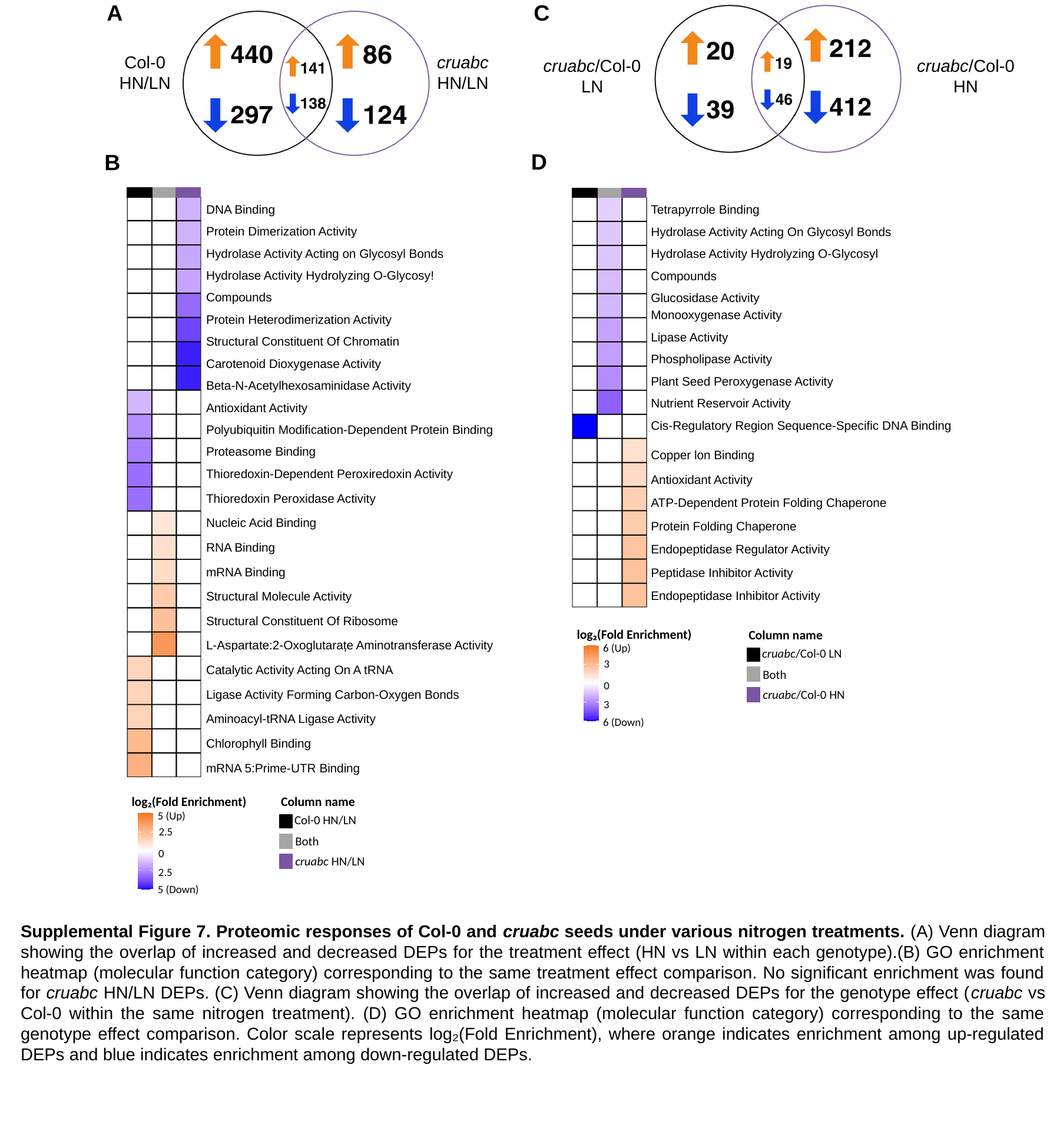

A
C
cruabc/Col-0
LN
cruabc/Col-0
HN
Col-0
HN/LN
cruabc
HN/LN
D
B
DNA Binding
Protein Dimerization Activity
Hydrolase Activity Acting on Glycosyl Bonds
Hydrolase Activity Hydrolyzing O-Glycosy!
Compounds
Protein Heterodimerization Activity
Structural Constituent Of Chromatin
Carotenoid Dioxygenase Activity
Beta-N-Acetylhexosaminidase Activity
Antioxidant Activity
Polyubiquitin Modification-Dependent Protein Binding
Proteasome Binding
Thioredoxin-Dependent Peroxiredoxin Activity
Thioredoxin Peroxidase Activity
Nucleic Acid Binding
RNA Binding
mRNA Binding
Structural Molecule Activity
Structural Constituent Of Ribosome
L-Aspartate:2-Oxoglutarațe Aminotransferase Activity
Catalytic Activity Acting On A tRNA
Ligase Activity Forming Carbon-Oxygen Bonds
Aminoacyl-tRNA Ligase Activity
Chlorophyll Binding
mRNA 5:Prime-UTR Binding
Tetrapyrrole Binding
Hydrolase Activity Acting On Glycosyl Bonds
Hydrolase Activity Hydrolyzing O-Glycosyl
Compounds
Glucosidase Activity
Monooxygenase Activity
Lipase Activity
Phospholipase Activity
Plant Seed Peroxygenase Activity
Nutrient Reservoir Activity
Cis-Regulatory Region Sequence-Specific DNA Binding
Copper lon Binding
Antioxidant Activity
ATP-Dependent Protein Folding Chaperone
Protein Folding Chaperone
Endopeptidase Regulator Activity
Peptidase Inhibitor Activity
Endopeptidase Inhibitor Activity
log₂(Fold Enrichment)
6 (Up)
3
0
3
6 (Down)
Column name
cruabc/Col-0 LN
Both
cruabc/Col-0 HN
log₂(Fold Enrichment)
5 (Up)
2.5
0
2.5
5 (Down)
Column name
Col-0 HN/LN
Both
cruabc HN/LN
Supplemental Figure 7. Proteomic responses of Col-0 and cruabc seeds under various nitrogen treatments. (A) Venn diagram showing the overlap of increased and decreased DEPs for the treatment effect (HN vs LN within each genotype).(B) GO enrichment heatmap (molecular function category) corresponding to the same treatment effect comparison. No significant enrichment was found for cruabc HN/LN DEPs. (C) Venn diagram showing the overlap of increased and decreased DEPs for the genotype effect (cruabc vs Col-0 within the same nitrogen treatment). (D) GO enrichment heatmap (molecular function category) corresponding to the same genotype effect comparison. Color scale represents log₂(Fold Enrichment), where orange indicates enrichment among up-regulated DEPs and blue indicates enrichment among down-regulated DEPs.

## Slide 8
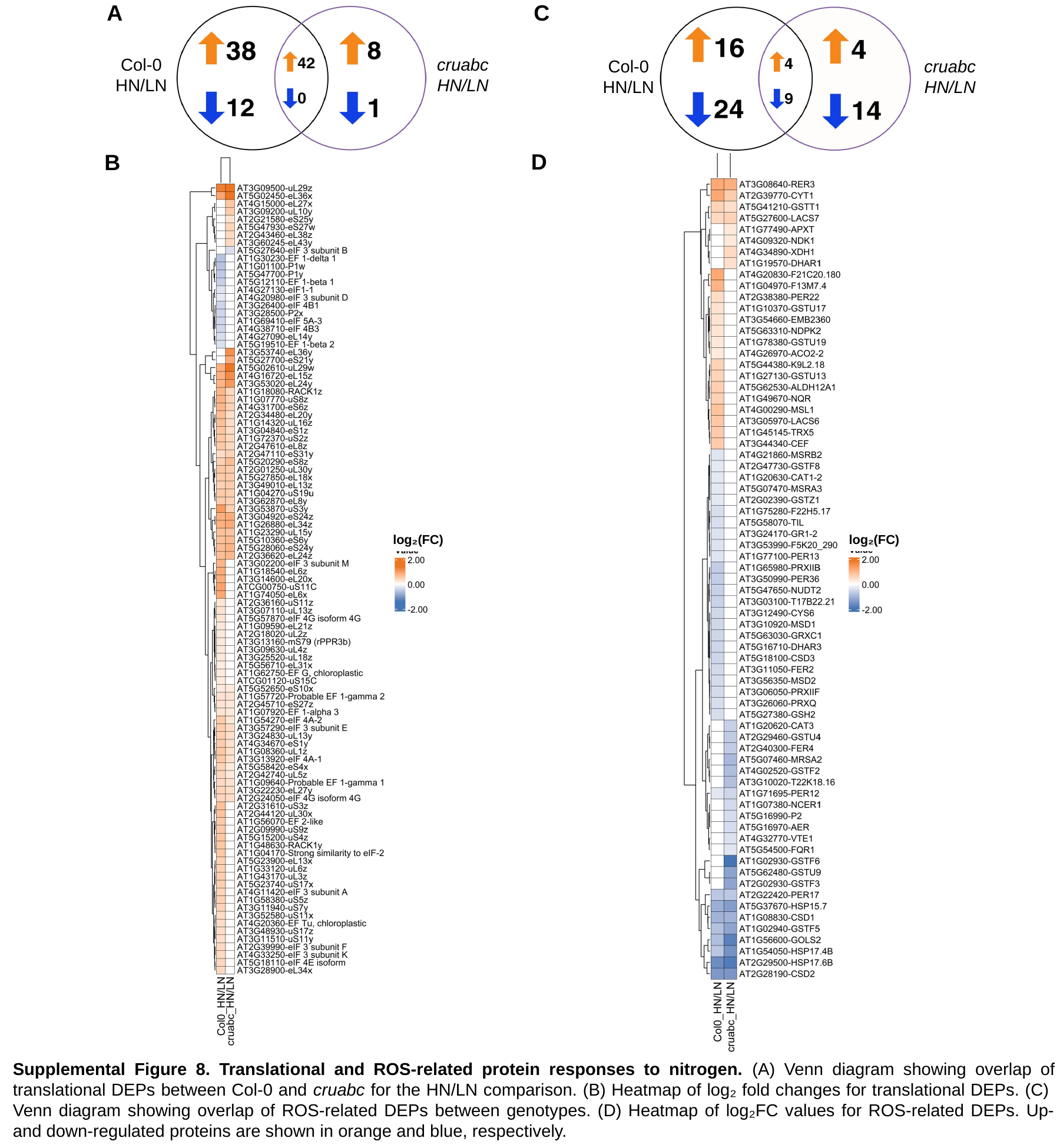

A
C
Col-0
HN/LN
cruabc
HN/LN
Col-0
HN/LN
cruabc
HN/LN
log₂(FC)
log₂(FC)
D
B
Supplemental Figure 8. Translational and ROS-related protein responses to nitrogen. (A) Venn diagram showing overlap of translational DEPs between Col-0 and cruabc for the HN/LN comparison. (B) Heatmap of log₂ fold changes for translational DEPs. (C) Venn diagram showing overlap of ROS-related DEPs between genotypes. (D) Heatmap of log₂FC values for ROS-related DEPs. Up- and down-regulated proteins are shown in orange and blue, respectively.
