# Supplementary material for: Environmental stress reveals new insights regarding proteome rebalancing in Arabidopsis thaliana seeds
Source: Plant J. 2026 Apr 20;126(2):e70881. doi: 10.1111/tpj.70881 (PMC13094328; doi:10.1111/tpj.70881)
Supplement: Supplementary file 7 — Table S1. Relative water content (RWC) of dry seeds harvested from Arabidopsis Col‐0 and cruabc plants subjected to various water and nitrogen treatments. The RWC was calculated as described in Material and Methods. A Duncan's multiple range test was used to compare the treatments, with same lowercase letters indicating no significant differences at the 5% level. Table S2. Comparison of protein‐bound amino acids (PBAAs) and free amino acids (FAAs) between the cruabc mutant and the Col‐0 wild type under full‐water (FW) and water‐deficit (WD) conditions. (A) Average PBAA levels and corresponding fold changes (cruabc/Col‐0, n = 5). (B) Average FAA levels and corresponding fold changes (cruabc/Col‐0, n = 4). Significance was determined using a two‐sample t‐test; bold values indicate amino acids with significant increases or decreases at P < 0.05. Table S3. Comparison of protein‐bound amino acid (PBAA) and free amino acid (FAA) composition between the cruabc mutant and the Col‐0 wild type under full‐water (FW) and water‐deficit (WD) conditions. (A) PBAA composition (%PBAA/total PBAA) and corresponding composition differences (%cruabc – %Col‐0). (B) FAA composition (%FAA/total FAA) and corresponding composition differences (%cruabc – %Col‐0). Significance was determined using a two‐sample t‐test; bold values indicate amino acids with significant increases or decreases at P < 0.05 (n = 5 for PBAAs, n = 4 for FAAs). Table S4. Comparison of protein‐bound amino acids (PBAAs) and free amino acids (FAAs) between the cruabc mutant and the Col‐0 wild type under low‐nitrogen (LN) and high‐nitrogen (HN) conditions. (A) Average PBAA levels and corresponding fold changes (cruabc/Col‐0, n = 5). (B) Average FAA levels and corresponding fold changes (cruabc/Col‐0, n = 4). Significance was determined using a two‐sample t‐test; bold values indicate amino acids with significant increases or decreases at P < 0.05. Table S5. Comparison of protein‐bound amino acid (PBAA) and free amino acid ( [file TPJ-126-0-s003.docx]

**Supplemental Table 1**. Relative water content (RWC) of dry seeds harvested from *Arabidopsis* Col-0 and *cruabc* plants subjected to various water and nitrogen treatments. The RWC was calculated as described in Material and Methods. A Duncan’s Multiple Range Test was used to compare the treatments, with same lower-case letters indicating no significant differences at the 5% level.

|  |  |  |
| --- | --- | --- |
| **Treatment** | **Seed Moisture (%)** | **Duncan test** |
| Col-0-FW | 5.91 ± 1.51 | a |
| Col-0-WD | 6.61 ± 1.18 | a |
| cruabc-FW | 5.51 ± 0.57 | a |
| cruabc-WD | 6.42 ± 0.75 | a |

**Supplemental Table 2**. Comparison of protein-bound amino acids (PBAAs) and free amino acids (FAAs) between the *cruabc* mutant and the Col-0 wild type under full-water (FW) and water-deficit (WD) conditions. (A) Average PBAA levels and corresponding fold changes (*cruabc* / Col-0, n = 5). (B) Average FAA levels and corresponding fold changes (*cruabc* / Col-0, n = 4). Significance was determined using a two-sample t-test; bold values indicate amino acids with significant increases or decreases at p < 0.05.

| **A** |  |  |  |  |  |  |  |  |  |  |  |  |  |  |  |  |
| --- | --- | --- | --- | --- | --- | --- | --- | --- | --- | --- | --- | --- | --- | --- | --- | --- |
|  | **PBAA levels (nmol/mg seed) and their ratio between *cruabc* and Col-0** | | | | | | | | | | | | | | | |
| **Treatment** | **Ala** | **Arg** | **Asx** | **Glx** | **His** | **Ile** | **Leu** | **Lys** | **Met** | **Phe** | **Pro** | **Ser** | **Thr** | **Tyr** | **Val** | **Total** |
| Col-0-FW | 97.34 | 61.19 | 84.12 | 169.30 | 35.62 | 72.82 | 100.00 | 51.67 | 16.85 | 52.87 | 91.26 | 86.99 | 80.59 | 34.65 | 75.80 | 1345.40 |
| cruabc-FW | 119.16 | 91.92 | 103.38 | 193.81 | 47.62 | 86.07 | 115.36 | 66.81 | 22.11 | 56.05 | 119.08 | 104.76 | 99.17 | 43.40 | 88.92 | 1614.83 |
| Fold | **1.22** | **1.50** | 1.23 | **1.14** | **1.34** | **1.18** | **1.15** | **1.29** | **1.31** | **1.06** | **1.30** | **1.20** | **1.23** | **1.25** | **1.17** | **1.20** |
| Col-0-WD | 149.97 | 107.74 | 149.38 | 231.51 | 50.55 | 117.95 | 160.28 | 66.82 | 24.42 | 58.49 | 140.30 | 131.01 | 123.53 | 55.68 | 116.88 | 1946.98 |
| cruabc-WD | 136.42 | 119.33 | 155.24 | 211.64 | 50.44 | 102.57 | 135.96 | 74.35 | 26.24 | 58.13 | 141.47 | 119.58 | 118.44 | 53.37 | 105.70 | 1869.17 |
| Fold | 0.91 | 1.11 | 1.04 | 0.91 | 1.00 | 0.87 | **0.85** | 1.11 | 1.07 | 0.99 | 1.01 | 0.91 | 0.96 | 0.96 | 0.90 | 0.96 |

| **B** |  |  |  |  |  |  |  |  |  |  |  |  |  |  |  |  |  |  |  |  |
| --- | --- | --- | --- | --- | --- | --- | --- | --- | --- | --- | --- | --- | --- | --- | --- | --- | --- | --- | --- | --- |
|  | **FAA levels (nmol/mg seed) and their ratio between *cruabc* and Col-0** | | | | | | | | | | | | | | | | | | | |
| **Treatment** | **Ala** | **Arg** | **Asn** | **Asp** | **Gln** | **Glu** | **His** | **Ile** | **Leu** | **Lys** | **Met** | **Phe** | **Pro** | **Ser** | **Trp** | **Thr** | **Tyr** | **Val** | **Cys** | **Total** |
| Col-0-FW | 0.66 | 1.50 | 3.02 | 2.18 | 0.27 | 8.86 | 0.33 | 0.19 | 0.34 | 0.20 | 0.19 | 0.68 | 0.40 | 1.09 | 2.49 | 0.41 | 0.26 | 0.64 | 0.03 | 23.70 |
| cruabc-FW | 1.44 | 4.92 | 18.74 | 5.69 | 1.34 | 11.09 | 0.98 | 0.50 | 0.80 | 0.49 | 0.43 | 0.79 | 1.12 | 2.74 | 4.69 | 1.18 | 0.90 | 2.34 | 0.06 | 60.24 |
| Fold | **2.18** | **3.29** | **6.21** | **2.61** | **5.03** | **1.25** | **3.02** | **2.67** | **2.39** | **2.50** | **2.31** | 1.17 | **2.84** | **2.52** | **1.88** | **2.88** | **3.43** | **3.68** | **1.80** | **2.54** |
| Col-0-WD | 1.63 | 3.83 | 12.41 | 5.81 | 1.74 | 10.78 | 0.78 | 0.41 | 0.65 | 1.17 | 0.29 | 1.19 | 7.66 | 2.37 | 1.08 | 2.00 | 0.52 | 1.72 | 0.06 | 56.09 |
| cruabc-WD | 3.97 | 6.56 | 30.51 | 11.56 | 3.91 | 13.73 | 1.54 | 1.20 | 1.35 | 7.42 | 0.52 | 1.35 | 14.07 | 5.06 | 4.84 | 7.52 | 1.58 | 6.53 | 0.23 | 123.44 |
| Fold | **2.44** | **1.71** | **2.46** | **1.99** | **2.25** | **1.27** | **1.98** | **2.94** | **2.08** | **6.35** | **1.83** | 1.13 | **1.84** | **2.13** | **4.48** | **3.77** | **3.05** | **3.80** | **3.81** | **2.20** |

**Supplemental Table 3**. Comparison of protein-bound amino acid (PBAA) and free amino acid (FAA) composition between the *cruabc* mutant and the Col-0 wild type under full-water (FW) and water-deficit (WD) conditions. (A) PBAA composition (%PBAA/total PBAA) and corresponding composition differences (%*cruabc* – %Col-0). (B) FAA composition (%FAA/total FAA) and corresponding composition differences (%*cruabc* – %Col-0). Significance was determined using a two-sample t-test; bold values indicate amino acids with significant increases or decreases at p < 0.05 (n = 5 for PBAAs, n = 4 for FAAs).

| **A** |  |  |  |  |  |  |  |  |  |  |  |  |  |  |  |
| --- | --- | --- | --- | --- | --- | --- | --- | --- | --- | --- | --- | --- | --- | --- | --- |
|  | **PBAA composition (%PBAA/TPBAA) and their difference between *cruabc* and Col-0** | | | | | | | | | | | | | | |
| **Treatment** | **Ala** | **Arg** | **Asx** | **Glx** | **His** | **Ile** | **Leu** | **Lys** | **Met** | **Phe** | **Pro** | **Ser** | **Thr** | **Tyr** | **Val** |
| Col-0-FW | 8.77 | 5.52 | 7.54 | 15.24 | 3.18 | 6.55 | 9.00 | 4.67 | 1.52 | 4.78 | 8.21 | 7.83 | 7.26 | 3.12 | 6.82 |
| cruabc-FW | 8.76 | 6.77 | 7.59 | 14.29 | 3.52 | 6.34 | 8.50 | 4.93 | 1.63 | 4.15 | 8.75 | 7.74 | 7.31 | 3.19 | 6.54 |
| Difference | 0.00 | **1.25** | 0.05 | **-0.96** | 0.34 | **-0.21** | **-0.50** | **0.26** | 0.11 | **-0.63** | **0.54** | -0.09 | 0.05 | 0.08 | **-0.28** |
| Col-0-WD | 8.91 | 6.40 | 8.86 | 13.73 | 3.01 | 7.00 | 9.52 | 3.97 | 1.45 | 3.49 | 8.31 | 7.78 | 7.33 | 3.30 | 6.94 |
| cruabc-WD | 8.47 | 7.43 | 9.60 | 13.24 | 3.15 | 6.36 | 8.44 | 4.63 | 1.64 | 3.64 | 8.74 | 7.44 | 7.36 | 3.32 | 6.56 |
| Difference | -0.44 | **1.03** | 0.74 | -0.48 | 0.14 | **-0.64** | **-1.09** | **0.66** | **0.19** | 0.15 | 0.42 | -0.34 | 0.03 | 0.01 | **-0.38** |

| **B** |  |  |  |  |  |  |  |  |  |  |  |  |  |  |  |  |  |  |  |
| --- | --- | --- | --- | --- | --- | --- | --- | --- | --- | --- | --- | --- | --- | --- | --- | --- | --- | --- | --- |
|  | **FAA composition (%FAA/TFAA) and their difference between *cruabc* and Col-0** | | | | | | | | | | | | | | | | | | |
| **Treatment** | **Ala** | **Arg** | **Asn** | **Asp** | **Gln** | **Glu** | **His** | **Ile** | **Leu** | **Lys** | **Met** | **Phe** | **Pro** | **Ser** | **Trp** | **Thr** | **Tyr** | **Val** | **Cys** |
| Col-0-FW | 2.80 | 6.31 | 12.69 | 9.21 | 1.13 | 37.38 | 1.38 | 0.79 | 1.41 | 0.83 | 0.78 | 2.84 | 1.67 | 4.57 | 10.55 | 1.73 | 1.11 | 2.68 | 0.14 |
| cruabc-FW | 2.38 | 8.17 | 30.98 | 9.46 | 2.23 | 18.50 | 1.64 | 0.82 | 1.33 | 0.81 | 0.71 | 1.31 | 1.85 | 4.53 | 7.87 | 1.94 | 1.49 | 3.88 | 0.10 |
| Difference | **-0.41** | **1.86** | **18.28** | 0.26 | **1.09** | **-18.88** | **0.26** | 0.04 | -0.08 | -0.02 | -0.07 | **-1.53** | 0.18 | -0.04 | **-2.69** | 0.21 | **0.39** | **1.20** | -0.04 |
| Col-0-WD | 2.88 | 6.92 | 21.91 | 10.38 | 2.95 | 19.57 | 1.41 | 0.72 | 1.16 | 2.01 | 0.52 | 2.16 | 13.56 | 4.26 | 2.00 | 3.51 | 0.94 | 3.05 | 0.11 |
| cruabc-WD | 3.14 | 5.35 | 24.75 | 9.32 | 3.13 | 11.29 | 1.26 | 0.93 | 1.10 | 6.05 | 0.43 | 1.08 | 11.39 | 4.11 | 3.98 | 6.06 | 1.26 | 5.20 | 0.18 |
| Difference | 0.26 | **-1.56** | 2.84 | -1.06 | 0.17 | **-8.28** | -0.15 | 0.22 | -0.06 | **4.04** | -0.09 | **-1.08** | -2.17 | -0.15 | **1.98** | **2.55** | **0.32** | **2.15** | 0.08 |

**Supplemental Table 4**. Comparison of protein-bound amino acids (PBAAs) and free amino acids (FAAs) between the *cruabc* mutant and the Col-0 wild type under low-nitrogen (LN) and high-nitrogen (HN) conditions. (A) Average PBAA levels and corresponding fold changes (*cruabc* / Col-0, n = 5). (B) Average FAA levels and corresponding fold changes (*cruabc* / Col-0, n = 4). Significance was determined using a two-sample t-test; bold values indicate amino acids with significant increases or decreases at p < 0.05.

| **A** |  |  |  |  |  |  |  |  |  |  |  |  |  |  |  |  |
| --- | --- | --- | --- | --- | --- | --- | --- | --- | --- | --- | --- | --- | --- | --- | --- | --- |
|  | **PBAA levels (nmol/mg seed) and their ratio between *cruabc* and Col-0** | | | | | | | | | | | | | | | |
| **Treatment** | **Ala** | **Arg** | **Asx** | **Glx** | **His** | **Ile** | **Leu** | **Lys** | **Met** | **Phe** | **Pro** | **Ser** | **Thr** | **Tyr** | **Val** | **Total** |
| Col-0-LN | 175.05 | 62.90 | 101.02 | 193.20 | 25.63 | 64.63 | 92.39 | 63.70 | 17.49 | 43.31 | 88.35 | 85.12 | 81.32 | 24.22 | 76.27 | 1179.36 |
| cruabc-LN | 163.55 | 60.51 | 83.62 | 188.04 | 25.69 | 58.33 | 81.51 | 67.28 | 18.32 | 42.28 | 90.08 | 79.68 | 75.89 | 23.44 | 73.36 | 1131.60 |
| Fold | 0.93 | 0.96 | **0.83** | 0.97 | 1.00 | **0.90** | **0.88** | **1.06** | 1.05 | 0.98 | 1.02 | 0.94 | 0.93 | 0.97 | 0.96 | 0.96 |
| Col-0-HN | 257.62 | 80.19 | 166.06 | 314.21 | 35.17 | 96.75 | 142.74 | 76.89 | 24.61 | 62.61 | 128.77 | 117.42 | 107.86 | 37.42 | 110.52 | 1758.84 |
| cruabc-HN | 257.86 | 83.10 | 156.12 | 296.88 | 38.38 | 84.44 | 123.69 | 89.02 | 25.92 | 61.62 | 133.06 | 107.72 | 106.01 | 38.59 | 96.42 | 1679.56 |
| Fold | 1.00 | 1.04 | 0.94 | 0.94 | **1.09** | **0.87** | **0.87** | **1.16** | 1.05 | 0.98 | 1.03 | **0.92** | 0.98 | 1.03 | 0.87 | 0.95 |

| **B** |  |  |  |  |  |  |  |  |  |  |  |  |  |  |  |  |  |  |  |  |
| --- | --- | --- | --- | --- | --- | --- | --- | --- | --- | --- | --- | --- | --- | --- | --- | --- | --- | --- | --- | --- |
|  | **FAA levels (nmol/mg seed) and their ratio between *cruabc* and Col-0** | | | | | | | | | | | | | | | | | | | |
| **Treatment** | **Ala** | **Arg** | **Asn** | **Asp** | **Gln** | **Glu** | **His** | **Ile** | **Leu** | **Lys** | **Met** | **Phe** | **Pro** | **Ser** | **Trp** | **Thr** | **Tyr** | **Val** | **Cys** | **Total** |
| Col-0-LN | 0.80 | 0.71 | 3.31 | 1.83 | 0.18 | 2.82 | 0.34 | 0.27 | 0.25 | 0.25 | 0.14 | 0.31 | 0.61 | 1.33 | 1.21 | 0.29 | 0.17 | 0.61 | 0.01 | 15.45 |
| cruabc-LN | 1.19 | 1.98 | 11.44 | 2.59 | 0.34 | 3.02 | 0.57 | 0.48 | 0.45 | 0.30 | 0.21 | 0.31 | 0.36 | 1.93 | 2.97 | 0.35 | 0.36 | 1.08 | 0.02 | 29.94 |
| Fold | 1.49 | **2.77** | **3.46** | **1.42** | 1.87 | 1.07 | 1.69 | **1.74** | **1.78** | 1.20 | **1.57** | 0.99 | 0.59 | 1.45 | **2.46** | 1.21 | **2.07** | 1.76 | 1.24 | **1.94** |
| Col-0-HN | 0.98 | 1.61 | 8.58 | 2.41 | 0.34 | 2.98 | 0.36 | 0.33 | 0.36 | 0.33 | 0.18 | 0.54 | 0.74 | 1.56 | 0.26 | 0.48 | 0.22 | 0.62 | 0.02 | 22.91 |
| cruabc-HN | 1.47 | 13.53 | 33.67 | 5.29 | 2.18 | 3.08 | 0.84 | 0.54 | 0.61 | 0.61 | 0.26 | 0.43 | 0.46 | 1.58 | 1.05 | 0.73 | 0.46 | 1.47 | 0.04 | 68.31 |
| Fold | **1.50** | **8.40** | **3.93** | **2.19** | **6.34** | 1.04 | **2.32** | **1.61** | **1.67** | **1.84** | **1.47** | **0.81** | **0.63** | 1.01 | **4.03** | **1.51** | **2.12** | **2.38** | **1.82** | **2.98** |

**Supplemental Table 5**. Comparison of protein-bound amino acid (PBAA) and free amino acid (FAA) composition between the *cruabc* mutant and the Col-0 wild type under low-nitrogen (LN) and high-nitrogen (HN) conditions. (A) PBAA composition (%PBAA/total PBAA) and corresponding composition differences (% *cruabc* – %Col-0). (B) FAA composition (%FAA/total FAA) and corresponding composition differences (% *cruabc* – %Col-0). Significance was determined using a two-sample t-test; bold values indicate amino acids with significant increases or decreases at p < 0.05 (n = 5 for PBAAs, n = 4 for FAAs).

| **A** |  |  |  |  |  |  |  |  |  |  |  |  |  |  |  |
| --- | --- | --- | --- | --- | --- | --- | --- | --- | --- | --- | --- | --- | --- | --- | --- |
|  | **PBAA composition (%PBAA/TPBAA) and their difference between *cruabc* and Col-0** | | | | | | | | | | | | | | |
| **Treatment** | **Ala** | **Arg** | **Asx** | **Glx** | **His** | **Ile** | **Leu** | **Lys** | **Met** | **Phe** | **Pro** | **Ser** | **Thr** | **Tyr** | **Val** |
| Col-0-LN | 14.86 | 5.34 | 8.57 | 16.39 | 2.18 | 5.48 | 7.83 | 5.41 | 1.48 | 3.68 | 7.50 | 7.23 | 6.89 | 2.05 | 6.39 |
| cruabc-LN | 14.44 | 5.35 | 7.39 | 16.62 | 2.27 | 5.16 | 7.20 | 5.95 | 1.62 | 3.74 | 7.97 | 7.04 | 6.71 | 2.07 | 6.48 |
| Difference | -0.41 | 0.01 | **-1.18** | 0.23 | 0.10 | **-0.33** | **-0.63** | **0.54** | **0.13** | 0.06 | 0.47 | -0.19 | -0.19 | 0.02 | 0.09 |
| Col-0-HN | 14.64 | 4.56 | 9.44 | 17.86 | 2.00 | 5.50 | 8.11 | 4.37 | 1.40 | 3.56 | 7.32 | 6.68 | 6.13 | 2.13 | 6.30 |
| cruabc-HN | 15.36 | 4.95 | 9.29 | 17.67 | 2.29 | 5.03 | 7.36 | 5.31 | 1.54 | 3.67 | 7.93 | 6.41 | 6.31 | 2.30 | 5.71 |
| Difference | 0.72 | **0.40** | -0.15 | -0.19 | **0.29** | **-0.47** | **-0.75** | **0.93** | **0.14** | 0.12 | **0.60** | **-0.26** | 0.18 | **0.17** | -0.58 |

| **B** |  |  |  |  |  |  |  |  |  |  |  |  |  |  |  |  |  |  |  |
| --- | --- | --- | --- | --- | --- | --- | --- | --- | --- | --- | --- | --- | --- | --- | --- | --- | --- | --- | --- |
|  | **FAA composition (%FAA/TFAA) and their difference between *cruabc* and Col-0** | | | | | | | | | | | | | | | | | | |
| **Treatment** | **Ala** | **Arg** | **Asn** | **Asp** | **Gln** | **Glu** | **His** | **Ile** | **Leu** | **Lys** | **Met** | **Phe** | **Pro** | **Ser** | **Trp** | **Thr** | **Tyr** | **Val** | **Cys** |
| Col-0-LN | 5.23 | 4.48 | 21.05 | 11.93 | 1.19 | 18.65 | 2.11 | 1.78 | 1.65 | 1.60 | 0.90 | 2.04 | 3.72 | 8.57 | 8.14 | 1.84 | 1.14 | 3.92 | 0.08 |
| cruabc-LN | 4.00 | 6.18 | 37.86 | 8.82 | 1.13 | 10.46 | 1.86 | 1.58 | 1.50 | 1.01 | 0.72 | 1.06 | 1.19 | 6.39 | 10.22 | 1.18 | 1.22 | 3.56 | 0.05 |
| Difference | **-1.23** | 1.70 | **16.82** | **-3.11** | -0.05 | **-8.19** | -0.24 | -0.20 | -0.15 | **-0.60** | -0.17 | **-0.99** | **-2.53** | **-2.18** | 2.08 | **-0.66** | 0.08 | -0.36 | -0.02 |
| Col-0-HN | 4.30 | 7.04 | 37.01 | 10.59 | 1.51 | 13.16 | 1.59 | 1.48 | 1.61 | 1.46 | 0.78 | 2.38 | 3.18 | 6.88 | 1.15 | 2.10 | 0.96 | 2.73 | 0.10 |
| cruabc-HN | 2.16 | 19.83 | 49.28 | 7.74 | 3.19 | 4.52 | 1.23 | 0.79 | 0.89 | 0.89 | 0.38 | 0.64 | 0.68 | 2.31 | 1.54 | 1.06 | 0.67 | 2.16 | 0.06 |
| Difference | **-2.15** | **12.79** | **12.27** | **-2.84** | **1.68** | **-8.65** | **-0.36** | **-0.69** | **-0.72** | **-0.56** | **-0.40** | **-1.74** | **-2.50** | **-4.57** | 0.39 | **-1.04** | **-0.29** | **-0.57** | **-0.04** |
